# Host iron deficiency protects against *Plasmodium* infection and drives parasite molecular reprofiling

**DOI:** 10.1126/sciadv.aeb0828

**Published:** 2026-02-27

**Authors:** Danielle Clucas, Cavan Bennett, Rebecca Harding, Anne Pettikiriarachchi, Andrew Baldi, Louise M. Randall, Ryan Steel, Ronan Mellin, Melissa Hobbs, Sabrina Caiazzo, Martin N. Mwangi, Katherine L. Fielding, Peter F. Hickey, Tracey M. Baldwin, Daniela Amann-Zalcenstein, Samantha J. Emery-Corbin, Glory Mzembe, Ernest Moya, Sabine Braat, Aaron Jex, Ayse Y. Demir, Hans Verhoef, Kamija S. Phiri, Beverley-Ann Biggs, Wai-Hong Tham, Justin A. Boddey, Sant-Rayn Pasricha, Ricardo Ataíde

**Affiliations:** ^1^ Infection and Global Health, The Walter and Eliza Hall Institute of Medical Research, Parkville, Victoria, Australia.; ^2^ Department of Medical Biology, The University of Melbourne, Parkville, Victoria, Australia.; ^3^ Diagnostic Haematology, The Royal Melbourne Hospital, Melbourne, Victoria, Australia.; ^4^ The Micronutrient Forum, Healthy Mothers Healthy Babies Consortium, Washington, DC, USA.; ^5^ Advanced Biology and Technology Division, The Walter and Eliza Hall Institute of Medical Research, Parkville, Victoria, Australia.; ^6^ Monash Proteomics and Metabolomics Platform, Department of Biochemistry and Molecular Biology, Monash Biomedicine Discovery Institute, Monash University, Clayton, VIC, Australia.; ^7^ Training and Research Unit of Excellence, Blantyre, Malawi.; ^8^ Department of Infectious Diseases, Peter Doherty Institute, University of Melbourne, Melbourne, Victoria, Australia.; ^9^ School of Population and Global Health, University of Melbourne, Melbourne, Victoria, Australia.; ^10^ Laboratory for Clinical Chemistry and Haematology, Meander Medical Centre, Amersfoort, Netherlands.; ^11^ Division of Human Nutrition and Health, Wageningen University, Wageningen, Netherlands.; ^12^ School of Public and Global Health, Kamuzu University of Health Sciences, Blantyre, Malawi.; ^13^ Research School of Biology, The Australian National University, Canberra, ACT, Australia.

## Abstract

Iron deficiency, anemia, and *Plasmodium* infection are global health challenges with overlapping geographical distributions, particularly affecting pregnant women in Africa, yet the mechanisms underlying their interactions remain poorly understood. We used a multilayered approach combining clinical data from Malawian pregnant women (*n* = 711) in the REVAMP trial, a genetic mouse model [*Tmprss6*-knockout (KO)], and in vitro *Plasmodium falciparum* cultures to clarify iron-malaria associations. Iron deficiency was associated with 50% reduced *P. falciparum* parasitemia in pregnant women [95% CI (30 to 64%), *P* < 0.0001], while iron-deficient mice exhibited improved survival against *P. berghei* (median 15.5 days versus 7.0 days for WT mice) and protection from cerebral malaria (83% versus 17% survival). Iron chelation substantially changed the transcriptomic and proteomic profile of cultured *P. falciparum* parasites. Intravenous iron supplementation did not increase parasitemia when coupled with malaria prevention. These findings demonstrate that iron deficiency protects against *Plasmodium* infection and support World Health Organization recommendations for iron supplementation in malaria-endemic regions when combined with adequate malaria prevention strategies in place.

## Teaser

Iron deficiency across three different biological systems shows protection against malaria.

## INTRODUCTION

Iron deficiency anemia and *Plasmodium* infection remain critical global health problems with substantial overlap in their geographical distribution. In 2021, an estimated 1.92 billion people worldwide experienced anemia, while approximately 247 million cases of malaria and 619,000 malaria-related deaths were reported in the same year (*1*, *2*). Across much of sub-Saharan Africa, both iron deficiency and *Plasmodium* infection coexist as substantial public health challenges, with anemia commonly resulting from either condition (*3*–*5*).

These conditions appear to interact in complex ways that complicate public health interventions. There are concerns that iron supplementation might increase susceptibility to *Plasmodium* infection. These concerns stem from evidence such as a large randomized controlled trial (RCT) of ~24,000 Tanzanian children in a malaria-endemic area, which demonstrated an increased risk of hospitalization, death, and malaria-specific hospitalization in children receiving oral iron compared to those receiving placebo (*6*). In addition, observational evidence suggests that iron deficiency may protect against malaria in children. For example, in a cohort of 785 Tanzanian children followed for up to 3 years, iron-deficient (ID) children had lower odds of subsequent parasitemia and reduced malaria-associated mortality (*7*).

The biological mechanisms underlying these interactions are multifaceted. *Plasmodium* infection may induce iron deficiency through several pathways, including hemolysis (*8*), infection-induced hepcidin up-regulation (*9*), and impaired iron absorption, while, simultaneously, iron deficiency may modify host susceptibility to the parasite (*10*–*12*). In vitro studies have demonstrated reduced *Plasmodium falciparum* growth and invasion in red blood cells (RBCs) from anemic participants, a protective effect that was reversed when iron supplementation was administered (*8*, *13*–*15*).

Pregnant women represent a particularly vulnerable population for both iron deficiency and *Plasmodium* infection (*16*, *17*). Approximately one-third of pregnancies in moderate and high malaria transmission countries in the African region are exposed to malaria. A similar proportion of pregnant women globally experience anemia (*18*), with half of these anemia cases expected to be iron responsive (*19*). Both anemia and malaria during pregnancy are associated with adverse maternal and neonatal outcomes, including low birth weight, which increases the risk of neonatal and infant mortality (*17*, *20*–*23*). Iron supplementation has been linked to increases in birth weight (*24*). These complex risk-benefit considerations underlie the World Health Organization’s (WHO) current recommendation for universal iron supplementation for pregnant women in areas where anemia prevalence exceeds 40%, many of which are also malaria endemic (*25*).

Despite these recommendations, notable knowledge gaps remain regarding the safety and efficacy of iron interventions in malaria-endemic regions, particularly for pregnant women. Previous studies have been limited by challenges in accurately diagnosing iron deficiency during inflammation, confounding by malaria prevention measures, and difficulty isolating iron status’ specific effects on *Plasmodium* infection from other factors (*26*–*28*).

In this study, we used a multisystem approach to clarify the association between iron deficiency and risk of malaria infection. First, we examined clinical data from the REVAMP clinical trial (*29*) in Malawian anemic pregnant women to assess the relationship between iron status and *P. falciparum* parasitemia detected by ultrasensitive polymerase chain reaction (PCR). Second, we used a genetic mouse model of iron deficiency [*Tmprss6-*KO, a gene involved in the systemic regulation of iron (*30*, *31*)], which allowed us to isolate the effect of iron deficiency on the progression of *Plasmodium* infection. Last, we explored the direct effects of iron chelation on *P. falciparum* parasites in vitro through transcriptomic and proteomic analyses. This comprehensive approach allowed us to test our hypothesis that iron deficiency confers protection against *Plasmodium* infection through direct effects on parasite metabolism, which would have implications for the safety of iron supplementation in malaria-endemic regions.

## RESULTS

### Sample characteristics

**Fig. 1.**
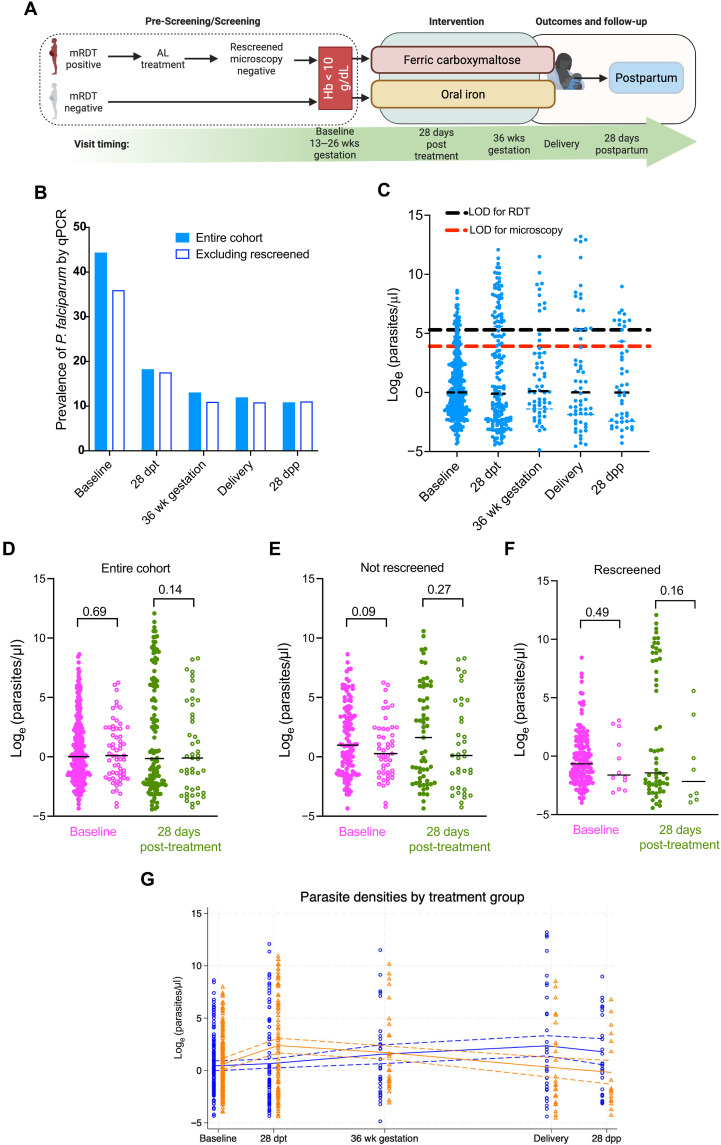
Trial cohort and *P. falciparum* prevalence and parasite density. (**A**) Flowchart depicting participant enrollment and sample collection timeline in the REVAMP trial. Women who tested positive by malaria rapid diagnostic test (RDT) at prescreening received AL treatment and were rescreened after at least 1 week before enrollment. Created with Biorender.com. (**B**) Prevalence of *P. falciparum* infection by ultrasensitive qPCR across study time points. All women (blue) or excluding those who were malaria RDT positive at screening, treated with AL and enrolled after microscopy-negative slide (rescreened) (white). (**C**) Parasite densities (parasites/μl) in qPCR-positive samples across study time points. Box plots show median, IQR, and range. The theoretical limits of detection (LOD) for RDT (black) and microscopy (red) are shown on the graph. (**D**) Parasite densities stratified by iron status [iron-replete (IR), full circles; iron-deficient (ID), empty circles] at baseline (magenta) and 28 days post-treatment (green) for all women. (**E**) Parasite densities stratified by iron status (IR, full circles; ID, empty circles) at baseline (magenta) and 28 days post-treatment (green) excluding those who were malaria RDT positive at screening, treated with AL and enrolled after microscopy-negative slide (rescreened). (**F**) Parasite densities stratified by iron status (IR, full circles; ID, empty circles) at baseline (magenta) and 28 days post-treatment (green) in those who were rescreened. (**G**) Parasite densities stratified by treatment group [IV iron (orange) versus oral iron (blue)] across time points. Lines represent median values with 95% confidence intervals. Dpt, days post treatment; wk., weeks; dpp, days postpartum. *P* values represent two-sample *t* tests (D) and (E) or Mann-Whitney (F). (A) was created in BioRender. R. Ataíde (2025) https://BioRender.com/k3e6352.

We evaluated a total of 3141 samples, collected from 711 women in Zomba, Southern Malawi, across five time points during the trial (Fig. 1A and table S1). All enrolled women were anemic by capillary hemoglobin (Hb), with Hb < 10 g/dl. Cohort characteristics were similar between time points, despite not all participants having samples at all time points (table S1). At enrollment, 55.9% (358 of 641) of women with available samples were primigravid and there was a relatively high percentage of women with HIV infection (14.2%; 90 of 634), which was expected in this setting. Iron deficiency and inflammation were common, with 39.1% (245 of 626) of women being ID and 52.7% (330 of 626) being inflamed. The proportion of women with iron deficiency at baseline increased with severity of anemia (table S2).

### *P. falciparum* infection by malaria RDT at enrollment

One-third (33.1%; 212 of 641) of the women included in this study were malaria positive at prescreening by rapid diagnostic test (RDT) performed on a finger-prick sample. These women were treated with artemether-lumefantrine (AL), rescreened (no sooner than 1 week after AL treatment) and malaria-free by malaria microscopy slide (Fig. 1A and table S1). Once enrolled, all women had a venous sample taken, which was subsequently used to repeat the malaria RDT, 1.4% (9 of 627) of samples had a venous sample positive RDT result.

### *P. falciparum* infection by qPCR

We assessed the prevalence of *P. falciparum* on venous whole blood at all time points by ultrasensitive quantitative PCR (qPCR) (Fig. 1B). A total of 284 of 641 (44.3%) of samples were *P. falciparum* positive by qPCR at enrollment. Excluding from this analysis all the women who were malaria RDT positive at screening and allowed to enter the trial after being rescreened (after AL treatment and with possible dead parasites or parasite DNA still in circulation) that prevalence dropped to 35.9% (154 of 429) (Fig. 1B). As all women who were not rescreened entered the trial after a negative malaria RDT, this shows that a high proportion of *P. falciparum* infections go undetected by RDT at this time point in pregnancy in this area of Malawi. *P. falciparum* infections during pregnancy (and even at delivery) can often be missed by conventional RDT or even microscopy (*32*, *33*). After enrollment, and receipt of the antimalarial sulfadoxine-pyrimethamine (IPTp-SP), the overall prevalence of positive qPCR for *P. falciparum* dropped to 18.2% (120 of 659) at 28 days posttreatment and 11.9% (46 of 386) at delivery (Fig. 1B). The prevalence was similar between those who entered the trial directly or after rescreen. The overall prevalence of *P. falciparum–*positive qPCR remained at 10.8% (32 of 295) after delivery and into the 28 days postpartum time point. It is worth noting that 81 of 284 (28.5%) of women with a positive qPCR result at baseline were also qPCR positive 28 days posttreatment. We were not able to assess if these were persisting infections or new infections. At baseline, parasite densities in those who were qPCR positive, were expectedly low [median interquartile range (IQR) 1.01 (0.22 to 9.48) parasites/μl] and remained so throughout all time points (Fig. 1C). Of those enrolled with a negative RDT (not rescreened), 11.6% (20 of 171) were positive by qPCR, possibly representing RDT false negatives (fig. S3, blue dots). Those who entered the study with a microscopy-negative slide after rescreening but with a positive qPCR may have had true submicroscopic infections, or likely had circulating dead parasites or parasite DNA (fig. S3, red dots).

### Impact of iron status on prevalence and density of parasitemia

We sought to understand the associations between iron status and *P. falciparum* infection and parasitemia. In the REVAMP women, at baseline, iron deficiency, measured as serum ferritin <15 μg/liter or ferritin <30 μg/liter if C-reactive protein (CRP) >5 mg/liter, was associated with a 63% reduction in the probability of being *P. falciparum* qPCR positive [95% confidence interval (CI; 51 to 73%)] (Table 1 and table S3). This association remained strong when adjusted for gestational age at baseline, gravidity status, HIV, rescreened + AL treatment, maternal age, education, income source, and religion (50% reduced risk, 30 to 64%) (Table 1). This association was maintained when excluding the women who were rescreened [55% adjusted reduced prevalence, 95% CI (34 to 69%)] (Table 1 and table S4).

**Table 1.** Comparison by ID/IR at baseline and qPCR positivity. ID indicates serum ferritin <15 μg/liter or ferritin <30 μg/liter if C-reactive protein >5 mg/liter.

	ID at baseline		IR at baseline		Unadjusted*		Adjusted*	
PCR positive	*N*	*n*/*N* (%)	*N*	*n*/*N* (%)	Effect size (95% CI)	*P* value	Effect size (95% CI)	*P* value
Baseline*	245	53/245 (21.6)	381	225/381 (59.1)	0.37 (0.27–0.49)	<0.0001	0.50 (0.36–0.70)	<0.0001
28 days posttreatment†	263	31/263 (11.8)	380	84/380 (22.1)	0.53 (0.35–0.80)	0.0027	0.66 (0.40–1.08)	0.096
**Excluding those malaria RDT-positive at screening, treated with AL, and enrolled after microscopy-negative slide (rescreened)**								
Baseline*	225	44/225 (19.6)	191	105/191 (55.0)	0.36 (0.25–0.51)	<0.0001	0.45 (0.31–0.66)	<0.0001
28 days posttreatment†	242	27/242 (11.2)	189	47/189 (24.9)	0.45 (0.28–0.72)	0.0009	0.59 (0.34–1.01)	0.053

* Unadjusted: N/A; adjusted: gestational age at baseline, gravidity status, HIV, rescreened + AL treatment, maternal age, education, income source, and religion. Poisson regression model with robust error variance was fit.

† Unadjusted: treatment group; adjusted: treatment group, gestational age at baseline, gravidity status, HIV, rescreened + AL treatment, maternal age, education, income source, and religion.

We looked at the association of *P. falciparum* qPCR positivity at 28 days posttreatment, considering iron deficiency status at baseline (Table 1). The strength and direction of these associations were maintained after adjustment but did not reach statistical significance [prevalence reductions of 34% (−0.8%, 60%), and 41% (−0.1%, 66%), respectively] (Table 1). Sensitivity analysis, performed to include the WHO definition of iron deficiency (serum ferritin <15 μg/liter or ferritin <70 μg/liter if CRP > 5 mg/liter), revealed similar patterns (tables S3 and S5). We found similar strength and direction of associations when compared to the main analysis. In addition, we conducted sensitivity analysis of the association between qPCR positivity at 28 days posttreatment and iron status at baseline in only those women entering the trial with a negative qPCR result at baseline, thus assessing the risk of becoming infected (table S6). We found no evidence of an effect of iron status at baseline on the risk of becoming qPCR positive [risk reductions ID versus iron replete (IR): 5% (−119%, 59%), *P* = 0.91].

Overall quantification of parasitemia in those who were qPCR positive, revealed similar parasite densities irrespective of iron status at both enrollment [mean difference (95%CI) −0.16 (−0.91 to 0.60), *P* = 0.69] and 28 days posttreatment [−0.17 (−2.75 to 0.40), *P* = 0.14] (Fig. 1D). Similar analysis of parasitemia on those individuals not rescreened or in those coming into the trial after being rescreened revealed baseline iron status was not associated with parasite densities at baseline or at the 28 days post-treatment visit (Fig. 1, E and F). Sensitivity analysis of the parasite densities at 28 days posttreatment by iron status at baseline in those women with a qPCR-negative result at baseline revealed a higher median parasite density in IR versus ID women [19.60 (0.33 to 558.50) parasites/μl versus 3.27 (0.70 to 439.66) parasites/μl] (table S7). Parasitemia burden or prevalence at 28 days posttreatment in either ID or IR women was not associated with the degree of hemoglobin improvement from baseline (fig. S4).

### Impact of iron intervention on prevalence and density of parasitemia

We next investigated the effect of supplementation with intravenous (IV) iron at baseline compared to oral iron on the subsequent prevalence of parasitemia (Table 2). Treatment with IV iron was not associated with increased prevalence of parasitemia positivity by qPCR at any of the subsequent visits when compared to oral iron. Adjusting the analysis for iron status at previous visit and time between visits (among others) did not alter the risk of becoming qPCR positive. The same pattern was observed when excluding from the analysis those individuals who were enrolled after rescreening (Table 2).

**Table 2.** Comparison of qPCR positivity by treatment group (IV iron versus oral iron). Adjusted: ID at previous visit, anemia at previous visit, HIV status, rescreened + AL treatment, gravidity, maternal age, concurrent gestational age, and time between visits.

	IV iron		Oral iron		Unadjusted*		Adjusted*	
PCR positive	*N*	*n*/*N* (%)	*N*	*n*/*N* (%)	Effect size (95% CI)	*P* value	Effect size (95% CI)	*P* value
28 days posttreatment	334	65/334 (19.5)	325	55/325 (16.9)	1.15 (0.83–1.59)	0.40	1.14 (0.81–1.61)	0.46
36 weeks gestation	182	26/182 (14.3)	195	23/195 (11.8)	1.21 (0.72–2.05)	0.47	1.45 (0.77–2.74)	0.25
Delivery	191	17/191 (8.9)	195	29/195 (14.9)	0.60 (0.34–1.05)	0.072	0.56 (0.30–1.02)	0.059
28 days postpartum	138	10/138 (7.2)	157	22/157 (14.0)	0.52 (0.25–1.05)	0.070	0.52 (0.24–1.14)†	0.10
**Excluding those malaria RDT-positive at screening, treated with AL, and enrolled after microscopy-negative slide (rescreened)**								
28 days posttreatment	226	40/226 (17.7)	219	38/219 (17.4)	1.02 (0.68–1.53)	0.92	0.95 (0.62–1.44)	0.81
36 weeks gestation	117	13/117 (11.1)	131	14/131 (10.7)	1.04 (0.51–2.12)	0.91	1.86 (0.65–5.31)	0.25
Delivery	120	10/120 (8.3)	131	17/131 (13.0)	0.64 (0.31–1.35)	0.24	0.67 (0.31–1.45)	0.31
28 days postpartum	85	5/85 (5.9)	106	16/106 (15.1)	0.39 (0.15–1.02)	0.056	0.38 (0.14–1.08)†	0.070

* Poisson regression model with robust error variance was fit (by time point). The model estimates the direct effect, not total effect given ID at previous visit, and anemia at previous visit are mediators in the model.

† As “adjusted,” excluding concurrent gestational age.

An analysis of the parasite densities across all visits by treatment group revealed higher parasite densities in the IV iron group at 28 days posttreatment [*N*, median (IQR) *N* = 86, 5.66 (0.12 to 601.13)] compared to the oral iron group (*N* = 85, 0.36 (0.08 to 33.23) (Fig. 1G). However, this pattern was inverted at delivery and 28 days postpartum, with individuals in the IV iron group presenting reduced parasite densities (Fig. 1G).

### Genetic dissection of iron homeostasis in animal models of malaria

**Fig. 2.**
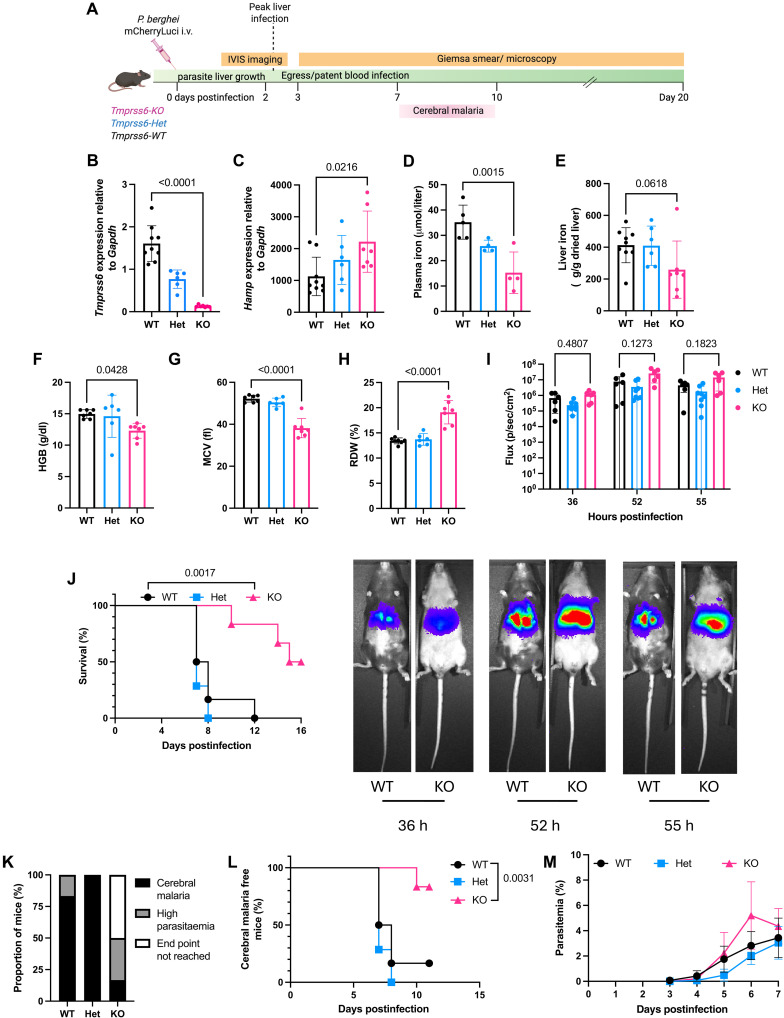
Iron deficiency in *Tmprss6*-KO mice provides protection against *P. berghei* infection. (**A**) Experimental design for *P. berghei* infection in C57BL/6 wild-type (WT), *Tmprss6*-heterozygous (Het), and *Tmprss6*-knockout (KO) mice. Mice reach cerebral malaria within 7 to 10 days postinfection. Created with Biorender.com (**B**) Hepatic *Tmprss6* mRNA expression in uninfected mice. (**C**) Hepatic hepcidin (*Hamp*) mRNA expression. (**D**) Plasma iron concentration. (**E**) Non-heme liver iron content. [(F) to (H)] Hematological parameters: (**F**) hemoglobin concentration, (**G**) mean corpuscular volume (MCV), and (**H**) red cell distribution width (RDW). (**I**) IVIS imaging of liver-stage infection showing parasite burden in all mice at indicated hours postinfection. Representative IVIS images of select mice are shown below. (**J**) Kaplan-Meier survival curves following *P. berghei* infection. (**K**) Percentage of mice succumbing to cerebral malaria (black), high parasitemia (gray), or not reaching humane end point (white). (**L**) Kaplan-Meier survival curve of mice succumbing to cerebral malaria. (**M**) Blood-stage parasitemia. Data represented as mean ± SD, *n* = 6 to 9 mice per group [(B), (C), and (E)], *n* = 4 to 5 (D), *n* = 6 to 7 [(F) to (H) and (J) to (M)], and *n* = 6 to 8 (I). (A) was created in BioRender. R. Ataíde (2025) https://BioRender.com/h6tqd73.

Interpreting the effects of iron status on *Plasmodium* risk in a field cohort is complex. Animal models of *Plasmodium* infection allow us to better dissect the timing and effect of iron status. To isolate the effects of iron status on *Plasmodium* infection and disease burden, we used *P. berghei* infection to induce liver and blood-stage infection, as well as cerebral malaria, in a mouse model of iron deficiency (*Tmprss6*-KO mice) (Fig. 2A).

Inactivation of *Tmprss6* causes an inability to suppress hepcidin expression, leading to reduced iron absorption and utilization, plasma iron depletion, and iron deficiency phenotype (*30*). Uninfected *Tmprss6*-KO mice had ablated hepatic *Tmprss6* mRNA expression (Fig. 2B), leading to increased hepcidin expression (Fig. 2C). *Tmprss6*-KO mice showed reduced plasma iron (Fig. 2D), and a non–statistically significant reduction in hepatic iron (Fig. 2E). As expected, *Tmprss6*-KO mice displayed iron restricted erythropoiesis with decreased hemoglobin concentrations and mean corpuscular volumes (MCVs) and increased red cell distribution width compared to wild-type (WT) mice (Fig. 2, F to H). *Tmprss6*-heterozygous (Het) mice have hepatic *Tmprss6* expression mid-way between WT and *Tmprss6*-KO animals with a complementary increase in hepcidin (*Hamp*) mRNA mid-way between WT and *Tmprss6*-KO (Fig. 2, B and C). However, *Tmprss6*-Het mice display iron and erythroid parameters almost identical to WT animals.

In vivo imaging system (IVIS) imaging of animals infected with *P. berghei* sporozoites expressing mCherry and luciferase revealed *Tmprss6*-KO mice had comparable intensity of liver infection to that of both WT and *Tmprss6*-Het mice from 36 to 55 hours postinfection (Fig. 2I). In this model of *P. berghei* infection, animals display cerebral malaria symptoms between days 7 and 10 postinfection. *Tmprss6*-KO mice experienced significantly improved survival compared to *Tmprss6*-Het and WT animals. The median survival for *P. berghei* infected *Tmprss6*-KO mice was 15.5 days compared with 7.5 days and 7 days for *Tmprss6*-Het and WT mice, respectively (*P* < 0.002) (Fig. 2J). Survival from cerebral malaria was also significantly improved in *Tmprss6*-KO mice (83% survival in *Tmprss6*-KO animals versus 17% WT; Fig. 2K) and time to succumb to cerebral malaria was delayed (median time to cerebral malaria: 10 days *Tmprss6*-KO versus 7 days WT; *P* < 0.005 Fig. 2I). However, parasitemia from day 3 to day 7 (the time point at which most WT and *Tmprss6*-Het mice succumbed to cerebral malaria), was similar between *Tmprss6*-KO and WT animals (Fig. 2M). Parasitemia was higher in the only WT mouse that survived cerebral malaria, when compared with the five surviving *Tmprss6*-KO mice (fig. S5).

### Direct effects of iron deprivation on *Plasmodium falciparum* in vitro

Having observed a protective effect of host iron deficiency on *Plasmodium* infection in both our clinical and animal models, we finally sought to establish whether iron chelation in parasite cultures functionally influences *Plasmodium* molecular profiles.

**Fig. 3.**
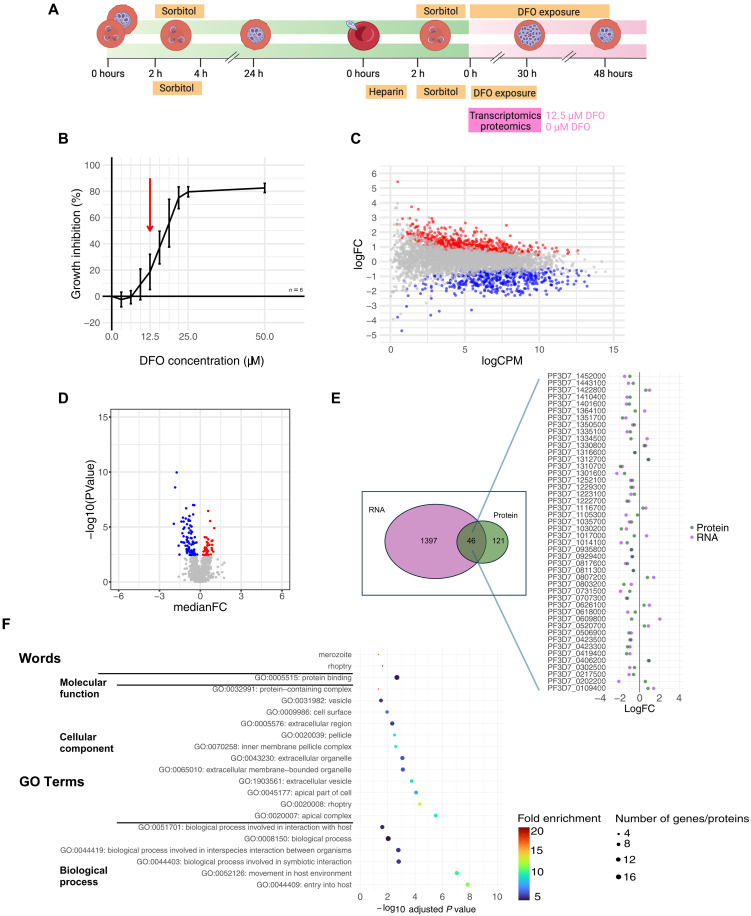
Iron chelation slows parasite development and induces extensive transcriptomic and proteomic changes in *P. falciparum*. (**A**) Experimental design for in vitro analysis of iron chelation on *P. berghei.* Created with Biorender.com (**B**) Dose-dependent growth inhibition of 3D7 *P. falciparum* parasites exposed to the iron chelator DFO for 48 hours (conducted as three technical replicates). (**C**) Volcano plot showing differentially expressed genes in parasites treated with 12.5 μM DFO for 30 hours [889 up-regulated (red), 508 down-regulated (blue) genes; log_2_ fold change ≥1, false discovery rate < 0.05]. (**D**) Volcano plot of differentially expressed proteins (40 up-regulated, 81 down-regulated; log_2_ fold change ≥1, false discovery rate < 0.05). (**E**) Overlap between differentially expressed genes and proteins, showing concordant and discordant regulation. (**F**) Enriched gene ontology (GO) biological process terms for the 46 differentially expressed gene-protein pairs, highlighting processes involved in host cell interaction and invasion. (B) Data represented as mean ± SD, *n* = 6. Proteomic and transcriptomic experiments were four biological replicates performed at least 1 week apart and subsequently processed together. (A) was created in BioRender. R. Ataíde (2025) https://BioRender.com/h6tqd73.

The antimalarial actions of iron chelators on blood-stage *Plasmodium* parasites have been previously identified but the full molecular effects of this chelation have not been thoroughly defined (*34*). We used the iron chelator desferrioxamine (DFO) in 3D7 *P. falciparum* blood-stage cultures followed by transcriptomic and proteomic analysis to investigate the effect of iron restriction (Fig. 3A). To confirm an antimalarial action of DFO, we first exposed in vitro cultures of 3D7 *P. falciparum* to DFO for 48 hours; a dose-dependent inhibition of parasite growth was evident (Fig. 3B). Similar to previous reports, we found the half-maximal inhibitory concentration for DFO to be 16.16 μM (95% CI 14.99 to 17.33) (*35*, *36*).

To characterize the effect of iron chelation on the transcriptome and proteome of the parasite, we performed RNA sequencing and quantitative proteomics on synchronous WT 3D7 *P. falciparum* cultures exposed to 12.5 μM of DFO for 30 hours from early ring-stage (4 to 6 hours post invasion, Fig. 3A), a concentration of DFO expected to affect parasite growth, without causing excessive parasite death that could render results difficult to interpret.

A total of 1397 genes were significantly differentially expressed (false discovery rate < 0.05) in cultures exposed to DFO for 30 hours compared with control parasites in media alone, of which 889 were up-regulated and 508 down-regulated (Fig. 3C). At the protein level, 121 proteins were differentially expressed in DFO-treated parasites compared with controls comprising 81 proteins significantly down-regulated and 40 up-regulated (Fig. 3D). Of these, 46 were differentially expressed at both the RNA and protein level. Most commonly this differential expression was in the same direction, 32 down-regulated in both and 9 up-regulated in both (Fig. 3E). Among the 46 gene/protein pairs differentially expressed in both analyses, 15 are associated with merozoite invasion (*37*), five are characterized as exported proteins (*38*), six are associated with the inner membrane complex (*39*, *40*), and four are associated with the food vacuole (*41*). Enrichment analysis performed for genes/proteins differentially expressed at both an RNA and protein level revealed enriched biological process gene ontology (GO) terms associated with interaction with the host and entry into host (Fig. 3F).

## DISCUSSION

Our study of the relationship between iron deficiency and malaria represents a unique approach, when compared to previous published studies. We collected data from three independent systems, clinical trial observations, animal models of iron deficiency, and molecular analyses, providing us with a breath of evidence across multiple biological contexts. Our multilayered approach to understanding the relationship between iron deficiency and *P. falciparum* infection has yielded findings across clinical, animal, and in vitro data, which consistently demonstrate a protective effect of iron deficiency. In Malawian pregnant women in the REVAMP trial, an RCT of IV iron versus standard of care oral iron in anemic women, iron deficiency at baseline in the second trimester, and before iron intervention, was associated with a 50% reduced probability of being qPCR positive for *P. falciparum*. In addition, iron deficiency at baseline was associated with a lower probability of *P. falciparum* parasitemia at 28 days postenrollment. These associations were maintained when looking exclusively at women who were malaria RDT-negative at prescreening, suggesting an effect that is independent from recent malaria treatment. Treatment with IV iron was not associated with an increased prevalence of parasitemia at any time point during the trial when compared with the standard of care, oral iron treatment. Complementing these clinical observations, our animal models using ID *Tmprss6-*KO mice exhibited improved survival and reduced cerebral malaria following *P. berghei* infection. Last, in vitro cultures using 3D7 parasites demonstrated antimalarial effects at the molecular level with iron chelation resulting in substantial inhibition of parasite growth and substantial changes in the transcriptome and proteome of *P. falciparum*. Together, our data all support the protective role of iron deficiency against *Plasmodium* infection and disease progression.

Previous studies in pregnant women have been contradictory about the protective effects of iron deficiency (*42*, *43*). Some of that contradiction may be derived from the definition of iron deficiency used in any particular study, as well as from the underlying characteristics of the women; however, a protective association of iron deficiency against *Plasmodium* infection is consistently observed when using ferritin as a biomarker of iron stores (*43*). The strong protective association we observed for maternal peripheral infections in the second trimester [50% reduced risk, 95% CI (30 to 64%), *P* < 0.0001] is of similar magnitude to the one found in a case-control study conducted in a similar area of Malawi in which placental malaria infections (measured at delivery) were found to be less frequent in women who were ID [odds ratio (OR) 0.4 (0.2 to 0.8), *P* = 0.01] (*44*). Also, a secondary analysis of an antimalarial RCT conducted in Papua New Guinea, where the mean hemoglobin at enrollment during the second trimester was 9.7 g/dl, found women who were ID at enrollment were 50% less likely to be *Plasmodium* positive by microscopy [adjusted OR, 0.50 (0.38 to 0.66), *P* < 0.001] (*28*). While most studies make concurrent observations of iron deficiency and *Plasmodium* infection, or look at association between time points that are extremely far apart (second trimester versus delivery), and are thus unable to determine the direction of the association, we determined the influence of iron deficiency at baseline with risk of *Plasmodium* infection 28 days after. Although all women in our cohort received iron treatment (either IV or oral) the iron utilization over 4 weeks would still be different between ID and replete women. In women overall, as well as excluding those women who received AL treatment before entering the study, iron deficiency at baseline was still more likely to protect from *Plasmodium* infection 28 days after (Table 1). Among women who were qPCR negative at baseline, iron status did not influence the risk of qPCR positivity at 28 days posttreatment, likely reflecting the stronger effect of being on trial (receipt of insecticide treated nets and antimalarial drugs) compared to the effect of iron status (table S4). While iron deficiency was protective overall, our analysis of IV iron versus oral iron supplementation showed no significant difference in subsequent parasitemia risk by qPCR across treatment groups. While women in both treatment groups showed responses to iron supplementation (though to a higher extent in the IV iron group), the degree of hemoglobin improvement from baseline to 28 days posttreatment was not associated with parasitemia burden or prevalence at 28 days post-treatment in either ID or IR women (fig. S4). This mirrors the findings of the main REVAMP trial (*29*) (which evaluated clinical malaria cases) and those of other iron supplementation RCTs in malaria endemic areas (*24*) and suggests that when appropriate malaria prevention measures are in place (all women received IPTp-SP or cotrimoxazole), iron supplementation, independently of the route of administration, can be given safely, supporting current WHO recommendations for universal iron supplementation in high-anemia-prevalence settings (*45*). However, the transiently higher parasite densities observed in the IV iron group at 28 days post-treatment highlight the need for increased vigilant malaria surveillance in the period immediately following iron repletion, a window of time during which women will be primed for erythropoiesis.

Our mouse model of iron deficiency, genetic KO of *Tmprss6*, a gene regulating systemic iron homeostasis (*30*, *31*), provides important mechanistic insights that are difficult to disentangle in human populations. In addition, our animal model of infection with sporozoites, replicates human infection, unlike previous studies that have bypassed liver-stage infection using infected red cells (*46*–*50*). After infection with *P. berghei* sporozoites, *Tmprss6*-KO mice displayed comparable liver infection intensity (Fig. 2I) despite reduced hepatic iron content (Fig. 2E), suggesting iron may not be critical for *Plasmodium* growth in the liver, or that minimal liver iron requirements for *Plasmodium* growth are below the reduction achieved in the model. Others have observed a protective effect of acute elevated hepcidin coupled with prior inflammation on liver infection burden caused by *P. berghei* sporozoites (*51*); however, the chronic elevated hepcidin in the absence of prior inflammation seen in our model did not exhibit the same liver-protection effect. Following *Plasmodium* egress from the liver, *Tmprss6*-KO animals displayed improved survival and protection from cerebral malaria. It has been shown that in vitro culture of 3D7 parasites in erythrocytes from ID donors result in lower parasitaemia when compared to 3D7 grown in erythrocytes from IR donors (*52*). The early development of blood-stage infection in *Tmprss6*-KO mice, in conditions where iron availability is restricted and erythropoiesis shows an ID phenotype (Fig. 2, D and F to H), did not differ (Fig. 2M) from WT mice. This mirrors the similar parasite densities observed in women who were ID compared to those who were IR in our clinical cohort (Fig. 1D). The low numbers of WT mice surviving past the cerebral malaria window in this model of infection (*N* = 1) do not allow us to make conclusions regarding a possible parasitemia control in *Tmprss6*-KO mice past day 7 (fig. S4). We observed a significant increase in survival (Fig. 2, J and K) and a delayed time to cerebral malaria in *Tmprss6*-KO mice (median 10 days versus 7 days in WT mice, *P* < 0.005), suggesting that iron restriction leading to iron-restricted erythropoiesis affects the pathogenic processes leading to severe disease manifestations (a finding that can have important clinical implications, as previously reported) (*53*). Both *Tmprss6*-KO mice (ID) and WT mice (IR) exhibited similar parasitaemia during the liver stage and the early blood stage before and during the onset of the cerebral malaria window. This appears to indicate that the differential iron status of these mice, which resulted in significantly different iron distributions, did not affect parasite replication, and thus the protective effect we observe in cerebral malaria is independent of the parasite burden. Possibly, it is a modification of the host pathophysiological response that is responsible for the observed protection from cerebral malaria, likely through changes in immune or endothelial cell activation, production of inflammatory factors, or blood-brain barrier integrity (*54*, *55*). This lack of effect on parasitemia in our mouse experiments mirrors what we observed in our clinical cohort, where iron deficiency was associated with less risk of infection, but not with parasite density.

Iron chelation with DFO is not only an in vitro tool but also a clinically relevant one (*53*). Our transcriptomic and proteomic analyses of *P. falciparum* cultured under iron-chelated conditions reveal the molecular adaptations that may underlie the parasite’s response to systemic iron restriction. Our transcriptome and proteome wide analysis of DFO-exposed *P. falciparum* parasite cultures identified substantial changes in mRNA and protein expression by iron chelation. We identified 1397 differentially expressed genes and 121 differentially expressed proteins in DFO-treated parasites, with 46 entities showing mostly concordant changes at both RNA and protein levels (41 of 46 pairs). Similar to the transcriptomic findings of 3D7 grown in ID erythrocytes (*52*), we identified changes in proteins and genes involved in merozoite invasion, protein export and RNA binding, and associated with the food vacuole, processes and organelles important for parasite survival, virulence, and nutrient acquisition. Despite the effects of DFO being almost certainly distinct from the possible protective effects of the microcytic hypochromic red cells resulting from host iron deficiency, the enrichment of GO terms related to host interaction and cell entry seems to complement the observations of a lower ability of *P. falciparum* to invade iron-depleted RBCs (*8*). The effects of iron restriction from the parasite and of host ID erythropoiesis could be additive and indicates that iron deficiency may protect by impairing the parasite’s ability to invade and establish infection within host cells, as well as impair parasite intra-erythrocytic replication.

This molecular in vitro evidence bridges our clinical observation of reduced infection prevalence in ID women with the improved survival seen in ID mice. These data highlight iron as an important nutrient for *P. falciparum* and indicate proteins of interest for future study.

While our manuscript focused primarily on the effects of iron on *Plasmodium* biology, there is an element of interaction between iron and immunity that represents another interface that can affect the relationship between iron deficiency and infection. Outside pregnancy, iron’s essential role in immune function is well established (*56*, *57*). Recent evidence shows that iron status influences antibody responses to vaccines (*58*) and may affect antibody functionality (*59*). In pregnancy, both the inflammatory and antibody response to *Plasmodium* are key elements that determine the course of the infection (*21*, *60*). In our pregnant clinical cohort, preexisting immunity (or lack of) from previous malaria exposures may interact with iron deficiency status. The reduced parasitemia risk we observed in ID women might reflect an optimal balance where modest reductions in adaptive immunity are offset by direct inhibitory effects on parasite growth and virulence. In addition, our transcriptomic and proteomic analyses under iron-restricted conditions revealed significant changes in parasite-exported proteins, as well as proteins essential for erythrocyte invasion and intraerythrocytic development. Many of these proteins are known to interact with the host immune system and modify infected erythrocyte surfaces, thereby affecting immunogenicity and cytoadherence properties (*61*, *62*). While these potential immune-related mechanisms provide additional context for our findings, they remain largely speculative and highlight important areas for future research.

Our findings have several important implications for public health policy and clinical practice. The observation that, early in pregnancy, women have a high proportion of low, submicroscopic parasitemias is prevalent across malaria in pregnancy studies and reinforces the need for active malaria surveillance and treatment strategies early in pregnancy, as these infections can result in poor pregnancy outcomes (*32*). Our data provide further evidence for the protective effect of iron deficiency on *Plasmodium* infection. The absence of increased infection risk in our IV iron treatment group (compared to standard of care oral iron) suggests that rapidly correcting iron deficiency can be done safely in anemic women when appropriate malaria control measures are in place. However, we reinforce the necessity, when implementing iron supplementation programs in malaria endemic areas, to include a period of active surveillance that considers possible increases in parasitaemia burden following IV iron. While correcting anemia remains a critical goal, particularly during pregnancy, iron repletion may transiently increase susceptibility to malaria highlighting the need for enhanced vigilance and possibly intensified malaria prevention during the period immediately following iron administration. Last, our animal and molecular findings suggest that under iron restriction conditions there are substantial transcriptomic and proteomic changes that are essential for parasite survival and pathogenicity. These can potentially be targeted as new therapeutics avenues.

Our study has several limitations that should be considered. In our clinical cohort, all women enrolled were anemic and received one form of iron supplementation. The REVAMP trial did not capture the distinction between positive mRDT tests representing *P. falciparum* infections (HRP2 pos or HRP2 neg) or potentially other species or mixed infections. While our models were adjusted for measured well-known confounders of the associations, estimates may be biased by the presence of unmeasured confounders. In addition, the numbers of women with longitudinal data available across the all trial did not allow for the necessary statistical power to model the interaction between iron deficiency and *Plasmodium* infection throughout. We also did not have enough infected women to evaluate the influence of new infections versus persistent infections in this cohort. We should also acknowledge that the data presented are not indicative of changes in parasite metabolism nor could we explore mechanistic ways in which iron deficiency protected these women. In our animal model, while the *Tmprss6*-KO approach offers several advantages for isolating the impact of iron deficiency, it does not perfectly recapitulate the complex and often multifactorial iron deficiency observed in pregnant human populations. Future studies could explore a range of iron deficiency models, including pregnancy models, with varying severity and mechanisms to better understand dose-response relationships. Our in vitro experiments were conducted using a single strain of *P. falciparum* and used DFO as an iron chelator, which may have effects beyond just iron restriction and may not replicate the iron-restricted erythropoiesis that is characteristic of iron deficiency. Alternative approaches using direct manipulation of parasite iron-related genes would strengthen the evidence regarding the role of iron in parasite biology.

In conclusion, our study demonstrates that iron deficiency protects against *Plasmodium* infection across clinical, animal, and molecular contexts. In anemic pregnant Malawian women, iron deficiency reduced *P. falciparum* parasitemia risk, while ID mice showed improved survival against cerebral malaria. Molecular analyses of DFO-exposed parasite cultures revealed that iron restriction significantly alters parasite transcriptomes and proteomes, particularly affecting invasion pathways. Future research should include the simultaneous analysis of transcriptomic and proteomic data from parasites collected from ID versus IR individuals as well as from in vitro cultures of long-term adapted parasites growing in ID or IR RBCs. Future research should also explore iron-dependent parasite metabolic pathways as potential therapeutic targets and optimize iron supplementation protocols to balance maternal health benefits with malaria risk in vulnerable populations.

## METHODS

### Study design and participants

Samples were derived from the REVAMP RCT (ANZCTR registration number ACTRN12618001268235) (*29*). REVAMP was an open-label RCT comparing a single dose of IV ferric carboxymaltose at enrollment to standard-of-care oral iron for the duration of pregnancy for anemia recovery in 862 anemic second-trimester pregnant women in Blantyre and Zomba districts in Malawi (a screening threshold of capillary hemoglobin <10.0 g/dl was used to target recruitment of clinically anemic women with venous hemoglobin <11.0 g/dl) (*29*). Oral or written informed consent was obtained from all women before screening, and written consent was obtained before being randomly assigned. All eligible pregnant women received insecticide treated nets, and intermittent preventative treatment with sulfadoxine/pyrimethamine (IPTp-SP, standard malaria prophylaxis during pregnancy) as per the Malawian national guidelines; HIV-positive women received cotrimoxazole (*63*). The trial received ethics approvals from the College of Medicine, University of Malawi, Malawi (P.02/18/2357), and The Walter and Eliza Hall Institute (WEHI), Australia (18/02).

### Procedures

Before enrollment in REVAMP (i.e., prescreening), women were screened for parasitemia by RDT (SD Bioline Malaria AG P.F/PAN, Standard Diagnostics, Inc.); positive RDT results included all tests with positive HRP2 and/or LDH test lines. RDT-positive women were excluded from enrollment and were treated with AL; however, if they met all other inclusion criteria, they could be rescreened after 1 week and enter the trial if they were malaria microscopy-negative (Fig. 1A). Venous blood was collected at enrollment (baseline, before randomization and treatment administration), at 28 days posttreatment, 36 weeks gestation, delivery, 28 days postpartum, and unscheduled visits when participants presented with illness during the trial (*64*). EDTA whole blood was banked for *P. falciparum* DNA analysis.

### Clinical laboratory measurements

Hemoglobin (Hb) concentration was measured on venous blood using a Sysmex XP-300 automated analyzer (Sysmex, Japan). Serum ferritin and CRP were measured at the Meander Medical Centre laboratory (accreditation number M040, EN ISO 15189:2012, Amersfoort, The Netherlands) using the Architect System (Abbott Ireland, Longford, Ireland). The lower limit of detection for ferritin was 3.1 μg/liter, and for CRP was 0.2 mg/liter.

### Parasitemia detection

DNA was extracted from 400 μl of EDTA whole blood using the QIAamp 96 Blood kit (Qiagen). DNA was also extracted from 200 μl of reconstituted WHO International Standard for *P. falciparum* DNA nucleic acid amplification techniques (NIBSC code: 04/176, National Institute for Biological Standards and Control, UK) (*65*). Pf-VarATS ultrasensitive PCR (*66*) was performed using published probe and primer sequences and Qiagen QuantiNova Probe PCR master mix, with master mix preparation and cycling conditions as per manufacturer recommendations. The lower limit of detection was 0.12 parasites/μl of blood. qPCR was performed on the LightCycler 480 II thermocycler (Roche) with samples run in triplicate.

### Animal models of iron deficiency

#### Animals and ethics

The iron deficiency model comprised *Tmprss6-*KO mice on a C57Bl/6 background (*31*). *Tmprss6*-Het littermates and WT animals were used as controls. Mice ranged from 9 to 35 weeks old. Both female and male mice were used. All animal experiments were conducted in accordance with the recommendations in the National Statement on Ethical Conduct in Animal Research of the National Health and Medical Research Council and under approved requirements set out by the WEHI of Medical Research Animal Ethics Committee, Melbourne, Australia (approvals 2017.031, 2019.013, 2021.064, and 2020.034). Animals were housed in specific pathogen–free conditions with access to standard chow (iron, 180 mg/kg) and water ad libitum.

#### Mosquito colony maintenance

*Anopheles stephensi* mosquitoes originally imported from Johns Hopkins School of Public Health, USA, were reared and maintained in the insectary at the WEHI, Melbourne, Australia according to standard methods. The mosquitoes were stored in BugDorm insect cages as mixed genders with an environment of 26° to 27°C, relative humidity of 70 to 80%, light/dark photoperiod 12:12 hours including 30-min ramping to imitate dawn and dusk. Mosquitoes were fed on reverse osmosis filtered water via cotton wicks and sugar cubes (sucrose-CSR).

#### P. berghei sporozoite production

To produce *P. berghei* sporozoites expressing mCherry and luciferase reporters (PbmcherryLuci, obtained from the Leiden University Medical Center) (*67*), BALB/c “donor” mice were infected via the intraperitoneal (i.p.) route with blood-stage parasites and 4 days later infected erythrocytes from the donor mice were transferred to naïve “acceptor” mice via i.p. or IV injection. Mice with ≥1% parasitemia and exhibiting exflagellation of microgametes by microscopy at 40× magnification were anesthetized with ketamine/xylazine via i.p. inoculation and individually placed on top of a single container of 50 female *An. stephensi* (3 to 5 days old) mosquitoes, allowing them to feed on mice for 15 to 30 min, after which any unfed mosquitoes were collected and discarded. Infected mosquitoes were maintained at 21°C with 80% humidity and 12:12-hour light:dark photoperiod with 30-min ramping. Midgut oocysts were determined at 14 days post-blood feeding and at 18 days post-blood feeding salivary glands were dissected from cold-anesthetized and ethanol killed mosquitoes into Schneider’s Insect Media (Sigma-Aldrich) pH 7.0 (*68*) and quantified in a hemocytometer counting chamber (Assistent, Neubauer improved) under phase at 400× magnification.

#### P. berghei infection and IVIS imaging

Mice were infected by IV injection of 6500 to 10000 PbmCherryLuci sporozoites (day 0). Mice were euthanized upon reaching any one of the following end points: signs of cerebral malaria (loss of self-righting reflex and hind-limb paresis) and >20% parasitemia; at least two of the following: weight loss >10%, hunched posture, piloerection, blanching of the tail and footpads, or palpable splenomegaly; or at day 20 postinfection (the experiment was terminated at day 16 postinfection as there were no surviving WT or HET mice). Liver-stage infection loads were assessed after injecting them with d-Luciferin in phosphate-buffered saline (PBS; IVISbrite, PerkinElmer), and imaging using an IVIS Lumina S5 (PerkinElmer) (*69*). WT and *Tmprss6*-Het mice were shaved around the abdomen before IVIS imaging to account for *Tmprss6*-KO mice having alopecia. Tail blood smears were performed daily from day 3 postinfection for blood-stage parasitemia assessment. Following euthanasia, blood was collected by cardiac puncture and livers were harvested for RNA extraction and liver iron quantitation.

#### Gene expression analysis

RNA (500 ng) was reverse transcribed to cDNA using the SensiFAST cDNA synthesis kit (Bioline) following the manufacturer’s protocol. Reverse transcription qPCR (RT-qPCR) using the SensiFAST SYBR No-ROX kit (Bioline) on a LightCycler 480 II thermocycler (Roche) was used to quantify gene expression levels. Primers and probes used for gene expression analysis and RT-qPCR can be found in table S8.

#### Animal specimen analysis

Liver taken into PBS and then snap frozen at −80°C was used to determine hepatic iron content as previously described (*70*). EDTA anticoagulated murine whole blood was analyzed on an Advia2120i for full blood count determination. Murine plasma iron was determined as previously described (*71*).

### In vitro effects of iron restriction on *P. falciparum*

#### Parasite culture and growth assays

Wild-type *P. falciparum 3D7* asexual blood stage parasites (originally obtained from David Walliker, University of Edinburgh) were cultured in Group O RhD+ red blood cells from healthy donors. Growth assays with DFO (Sigma-Aldrich) were set up using sorbitol synchronized ring-stage cultures with a starting parasitemia of ~1%, assessed by flow cytometry, at 1% hematocrit (HCT), and 50 μl volume. At 48 hours, parasites were stained with Hoechst and parasitemia analyzed by flow cytometry on a Verse flow cytometer (BD Biosciences, San Jose, USA). Flow cytometry data were analyzed using FlowJo software version 10.0.8 (Tree Star).

Samples for mass spectrometry (MS) and RNA sequencing were obtained from parasite cultures synchronized using a combination of serial sorbitol treatments and heparin exposure before being set up at early ring stage (4 to 6 hours postinvasion) with a ~1% starting parasitemia, 1% HCT, and total volume of 10 ml with concentrations of 0 and 12.5 μM of DFO. After 30 hours, cultures were treated with saponin to obtain parasite pellets which were frozen at −80°C for proteomic analysis or resuspended in 50 μl of RNAlater and stored at −20°C for RNA extraction and sequencing. Four biological replicates were performed at least 1 week apart, and all samples from the replicates were subsequently processed together.

#### Mass spectrometry

Parasite pellets were lysed in 8 M urea, 100 mM trisaminomethane Tris (pH 8.8), with protein concentrations determined and normalized by BCA assay (Pierce). Proteins were reduced (10 mM dithiothreitol), alkylated [(15 mM iodoacetamide (IAM)], and digested overnight with trypsin (1:100). Peptides were acidified, desalted using C18 tips (in house), vacuum-dried, and reconstituted in 2% acetonitrile (ACN) and 0.1% trifluoroacetic acid for liquid chromatography–MS analysis. MS was performed on an Orbitrap Fusion Lumos with FAIMS Pro interface (CVs: −40 and −60 V). MS1 scans were acquired at 120k resolution with AGC target 4 × 10^5^ (normalized AGC 100%) and max injection time (maxIT) of 50 ms. MS2 scans were acquired at 60k resolution with AGC target 1 × 10^4^ (normalized AGC 100%) and 50 ms maxIT. All spectra were acquired in positive mode with full-scan MS spectra scanning from mass/charge ratio (*m*/*z*) 300 to 1600 using a ±1.6 *m*/*z* isolation window and collision-induced dissociation (CID) at 35% collision energy. Peptide separation was performed by nanoflow reversed-phase high-performance liquid chromatography (Ultimate 3000 RSLC, Dionex) using an Acclaim PepMap C18 nano-trap column (75 μm by 2 cm) and analytical column (75 μm by 50 cm). The gradient was 3 to 23% buffer B over 29 min, 23 to 40% B in 10 min, 40 to 80% B in 5 min, held at 80% B for 5 min, then re-equilibrated at 3% B for 10 min. Buffer A is 0.1% FA and buffer B is 80% ACN and 0.1% FA. The MS proteomics data have been deposited to the ProteomeXchange Consortium via the PRIDE (*72*) partner repository with the dataset identifier PXD064348.

#### RNA sequencing

RNA was extracted using the Isolate II RNA mini kit (Bioline) as per the manufacturer’s protocol. RNA was quantified using the Qubit 2.0 HS RNA assay (Thermo Fisher Scientific) and the quality assessed using the 2200 TapeStation System High Sensitivity RNA Screen Tape (Agilent). After normalization to RNA concentrations of 10 ng/μl, sequencing was performed using an in-house mini-bulk sequencing protocol. In short, single-cell transcriptome libraries were generated by adapting the CelsSeq2 protocol (*73*) as follows: Samples were pooled after first strand cDNA synthesis, treated with Exonuclease 1 for 30 min, followed by a 1.2× bead clean-up. Second strand synthesis was performed using NEBNext Second Strand Synthesis module (NEB) in a final reaction volume of 20 μl and NucleoMag NGS Clean-up and Size select magnetic beads (Macherey-Nagel) were used for all DNA purification and size selection steps. Sequencing was performed on the Illumina NextSeq2000 instrument (San Diego, USA), using a 100 cycle kit.

#### Bioinformatic analysis

In brief, database searching for MS data was performed using MaxQuant version 1.6.10.43 (*74*). Each raw file was split into two mzXML files using FAIMS-MzXML-Generator for the two CVs used. We assigned the two CVs per raw file as fraction 1 (−40 CV) and fraction 2 (−60 CV) across all the files. The *P. falciparum* 3D7 isolate genome was downloaded from PlasmoDB (release 51) (*75*) and used for database searching. For RNA sequencing, expression was quantified by counting the number of unique molecular identifiers and reads mapped to each gene {*P. falciparum* 3D7 genome downloaded from PlasmoDB release 51, and spike-in transcripts [External RNA Controls Consortium (ERCC)]} using scPipe (v1.14.0) (*76*). Only exonic reads were included in the differential expression analysis.

#### In vitro analyses

Data analysis for MS was performed as previously outlined (*77*). Peptide intensities were summed, and nonunique peptides, reverse peptide decoys, and contaminants were removed. Unique peptides detected in a minimum of 18 samples were log transformed and quartile normalized using limma package version 3.46.0 in R (*78*), and missing values imputed using MSImpute using the v1 method (*79*). Differentially expressed peptides between conditions were identified using linear models with empirical Bayes moderated *t* statistics. Differentially expressed proteins were estimated using peptide set enrichment analysis in a gene set enrichment analysis framework (*80*). For RNA sequencing analysis, differential expression was performed using the negative binomial GLM framework workflow in edgeR (version 3.34.0), with enrichment analyses were performed in PlasmoDB. These identified GO (*81*) terms and metabolic pathways (from pathway databases, Kyoto Encyclopedia of Genes and Genomes (*82*) and MetaCyC (*83*).

### Statistical analysis

#### Clinical data

Iron deficiency was defined as ferritin <15 μg/liter, or <30 μg/liter if CRP was >5 mg/liter. Associations between iron status and *P. falciparum* infection and parasitemia at baseline and 28 days posttreatment, as well as the effect of supplementation with IV iron at baseline on the subsequent risk of parasitemia, were analyzed with Poisson regression models with a log link and robust error variance. Results are presented as point estimates and two-sided 95% CIs. Additional analyses included adjusted models for prespecified covariates with the use of directed acyclic graphs as presented in figs. S1 and S2 and sensitivity analysis excluding women who were malaria RDT positive at screening and were rescreened and deemed malaria microscopy-slide negative at enrollment. For analysis of parasite densities across time points by treatment group, we calculated median values with IQRs. Because of the skewed nature of the data, parasite densities were log transformed and groups were compared using appropriate statistical testing. Analyses were performed using Stata SE, version 18.0 (StataCorp, College Station, TX).

#### Animal models

Sample sizes for animal experiments were calculated on the basis of expected differences in survival between groups with 80% power at an alpha of 0.05, accounting for expected effect sizes based on preliminary data. Statistical testing of continuous variables was performed using ordinary one-way analysis of variance (ANOVA) with Dunnett’s correction for multiple comparisons (except Fig. 2E, where a Kruskal-Wallis test with Dunn’s correction for multiple comparisons was used) or two-way repeated-measures ANOVA with Dunnett’s correction for multiple comparisons. Survival analyses were performed using Gehan-Breslow-Wilcoxon tests. Statistical analyses were performed in Prism version 10.4.1 (GraphPad software).
