# Supplementary material for: Host iron deficiency protects against Plasmodium infection and drives parasite molecular reprofiling
Source: Sci Adv. 2026 Feb 27;12(9):eaeb0828. doi: 10.1126/sciadv.aeb0828 (PMC12947883; doi:10.1126/sciadv.aeb0828)
Supplement: Supplementary file 1 — Tables S1 to S8 Figs. S1 to S5 [file sciadv.aeb0828_sm.pdf]

Science Advances
AAAS

Supplementary Materials for

Host iron deficiency protects against *Plasmodium* infection and drives parasite molecular reprofiling

Danielle Clucas *et al.*

Corresponding author: Ricardo Ataíde, ataide.r@wehi.edu.au

Sci. Adv. **12**, eaeb0828 (2026)
DOI: 10.1126/sciadv.aeb0828

This PDF file includes:

Tables S1 to S8
Figs. S1 to S5

Supplementary Table 1. Baseline Characteristics for those with non-missing parasitemia across all visits

	Baseline	28 days post treatment	36 weeks gestation	Delivery	28 days postpartum
	N=641	N=659	N=377	N=385	N=295
Age (years)	22·0 (6·1)	22·0 (6·1)	22·6 (6·3)	22·7 (6·4)	22·9 (6·5)
Primigravid	358/641 (55·9%)	370/659 (56·1%)	196/377 (52·0%)	201/385 (52·2%)	148/295 (50·2%)
Gestational age (weeks)	22·0 (19·6-24·3)	22·0 (19·6-24·1)	22·4 (19·7-24·4)	22·1 (19·6-24·6)	22·4 (19·9-24·6)
Height (cm)	155·3 (6·5)	155·2 (6·5)	155·7 (6·7)	155·8 (6·4)	155·9 (6·9)
Weight (kg)	55·3 (8·2)	55·3 (8·2)	55·6 (8·3)	55·7 (8·1)	56·0 (8·1)
Body Mass Index (kg/m2) †	22·9 (3·0)	22·9 (3·0)	22·9 (3·0)	22·9 (2·9)	23·0 (2·9)
Religion ‡					
None	2/638 (0·3%)	2/656 (0·3%)	0/375 (0·0%)	0/383 (0·0%)	0/293 (0·0%)
Christian	438/638 (68·7%)	449/656 (68·4%)	256/375 (68·3%)	266/383 (69·5%)	201/293 (68·6%)
Muslim	193/638 (30·3%)	200/656 (30·5%)	118/375 (31·5%)	115/383 (30·0%)	91/293 (31·1%)
Other	5/638 (0·8%)	5/656 (0·8%)	1/375 (0·3%)	2/383 (0·5%)	1/293 (0·3%)
Education ‡					
None	2/620 (0·3%)	2/636 (0·3%)	1/364 (0·3%)	2/373 (0·5%)	1/284 (0·4%)
Lower Primary(1-5)	146/620 (23·5%)	146/636 (23·0%)	75/364 (20·6%)	73/373 (19·6%)	53/284 (18·7%)
Upper Primary(6-8)	269/620 (43·4%)	279/636 (43·9%)	153/364 (42·0%)	164/373 (44·0%)	119/284 (41·9%)
Lower Secondary(1-2)	83/620 (13·4%)	85/636 (13·4%)	57/364 (15·7%)	59/373 (15·8%)	49/284 (17·3%)
Upper Secondary(3-4)	107/620 (17·3%)	111/636 (17·5%)	71/364 (19·5%)	69/373 (18·5%)	56/284 (19·7%)
Tertiary	13/620 (2·1%)	13/636 (2·0%)	7/364 (1·9%)	6/373 (1·6%)	6/284 (2·1%)
Marital status ‡					
Single	106/638 (16·6%)	107/656 (16·3%)	66/375 (17·6%)	61/383 (15·9%)	53/293 (18·1%)
Married	519/638 (81·3%)	537/656 (81·9%)	302/375 (80·5%)	315/383 (82·2%)	237/293 (80·9%)
Widowed	3/638 (0·5%)	2/656 (0·3%)	2/375 (0·5%)	3/383 (0·8%)	2/293 (0·7%)
Divorced/Separated	10/638 (1·6%)	9/656 (1·4%)	5/375 (1·3%)	4/383 (1·0%)	1/293 (0·3%)
Others	0/638 (0·0%)	1/656 (0·2%)	0/375 (0·0%)	0/383 (0·0%)	0/293 (0·0%)
Income source ‡					
None	48/638 (7·5%)	47/656 (7·2%)	31/375 (8·3%)	32/383 (8·4%)	28/293 (9·6%)
Subsistence farming	131/638 (20·5%)	135/656 (20·6%)	70/375 (18·7%)	68/383 (17·8%)	48/293 (16·4%)
Large scale farming	2/638 (0·3%)	2/656 (0·3%)	1/375 (0·3%)	1/383 (0·3%)	1/293 (0·3%)
Employed	79/638 (12·4%)	80/656 (12·2%)	47/375 (12·5%)	50/383 (13·1%)	37/293 (12·6%)
Casual work for wages	212/638 (33·2%)	226/656 (34·5%)	118/375 (31·5%)	124/383 (32·4%)	87/293 (29·7%)
Business	159/638 (24·9%)	158/656 (24·1%)	104/375 (27·7%)	104/383 (27·2%)	89/293 (30·4%)
Other	7/638 (1·1%)	8/656 (1·2%)	4/375 (1·1%)	4/383 (1·0%)	3/293 (1·0%)
HIV positive ‡	90/634 (14·2%)	92/652 (14·1%)	55/372 (14·8%)	59/379 (15·6%)	44/291 (15·1%)
Malaria RDT positive ¶	9/627 (1·4%)	11/647 (1·7%)	4/369 (1·1%)	3/378 (0·8%)	3/291 (1·0%)
Re-screened post positive malaria RDT §	212/641 (33·1%)	214/659 (32·5%)	129/377 (34·2%)	134/385 (34·8%)	104/295 (35·3%)
Venous Hb (g/dL)	8·83 (1·21)	8·83 (1·20)	8·88 (1·22)	8·90 (1·22)	8·91 (1·25)
Anaemia status (venous)					
No (Hb≥ 11 g/dL)	30/638 (4·7%)	28/655 (4·3%)	20/375 (5·3%)	21/381 (5·5%)	18/294 (6·1%)
Mild (10g/dL ≤ Hb < 11 g/dL)	74/638 (11·6%)	77/655 (11·8%)	46/375 (12·3%)	46/381 (12·1%)	37/294 (12·6%)
Moderate (7 g/dL ≤Hb <10 g/dL)	494/638 (77·4%)	508/655 (77·6%)	286/375 (76·3%)	289/381 (75·9%)	222/294 (75·5%)
Severe (Hb <7 g/dL)	40/638 (6·3%)	42/655 (6·4%)	23/375 (6·1%)	25/381 (6·6%)	17/294 (5·8%)
Ferritin (µg/L)	31·40 (10·50-79·30)	28·90 (10·30-81·60)	30·00 (9·30-80·70)	27·90 (9·70-73·00)	27·65 (9·20-66·20)
C-reactive protein (mg/L)	5·35 (2·90-11·00)	5·20 (2·90-11·40)	5·50 (2·80-11·20)	5·40 (2·80-11·20)	5·65 (2·90-11·70)
Iron deficient ‖	245/626 (39·1%)	263/643 (40·9%)	158/375 (42·1%)	164/383 (42·8%)	132/294 (44·9%)
Iron deficient anaemia ‖	232/623 (37·2%)	250/639 (39·1%)	148/373 (39·7%)	151/379 (39·8%)	122/293 (41·6%)
Inflammation *	330/626 (52·7%)	334/643 (51·9%)	203/375 (54·1%)	203/383 (53·0%)	157/294 (53·4%)
Anaemia and inflammation *	313/623 (50·2%)	318/639 (49·8%)	191/373 (51·2%)	190/379 (50·1%)	145/293 (49·5%)

No./total no. (%) are counts and percentages Plus-minus values are means ±SD. RDT denotes rapid diagnostic test (for *Plasmodium* parasitaemia), Hb haemoglobin, and HIV human immunodeficiency virus.
† Body mass index is the weight in kilograms divided by the square of the height in meters.
‡ Religion, Education, Marital status, Income source, parity, gravidity and HIV status were self-reported.
§ If women met the anaemia criteria but had a positive RDT, they were treated for malaria as per local protocols and deferred from enrolment. Women were invited to be re-screened – no earlier than seven days later – and be enrolled if they met the eligibility criteria (in these cases, parasitemia was assessed using microscopy due to persistence of antigen detection via RDT).
¶ Malaria RDT positive based on confirmatory RDT testing by lab personnel on venous blood collected at enrolment
|| Iron deficient indicates serum ferritin<15ug/L or ferritin<30ug/L if C-reactive protein>5mg/L, and iron deficiency anaemia indicates Hb<11g/dL and serum ferritin<15µg/L or ferritin<30µg/L if C-reactive protein>5mg/L.
* Inflammation indicates C-reactive protein>5mg/L, and anaemia and inflammation indicates Hb<11.0g/dL and C-reactive protein>5mg/L.

Supplementary Table 2. Distribution of iron deficient women according to anaemia severity status

	No anemia at baseline	Mild anemia at baseline	Moderate anemia at baseline	Severe anemia at baseline
**Iron deficient at baseline**	14/42 (33.3%)	41/100 (41.0%)	274/638 (42.9%	34/60 (56.7%
**Iron replete at baseline**	28/42 (66.7%)	59/100 (59.0%)	364/638 (57.1%)	26/60 (43.3%)

n/N(%)

Iron deficient indicates serum ferritin <15 μ g/L or ferritin <30 μ g/L if C-reactive protein >5 mg/L.

Anemia status at baseline (based on venous Hb): No (Hb≥ 11 g/dL); Mild (10g/dL ≤ Hb < 11 g/dL); Moderate (7 g/dL ≤Hb <10 g/dL); Severe (Hb <7 g/dL)

*Venous Hb data are missing for 2 women who are iron deficient at base, and one woman iron replete at base.

Supplementary Table 3. Baseline Characteristics for those with non-missing parasitemia by iron status

	**Iron deficiency defined as Ferritin<15μg/L or ferritin<30μg/L if CRP>5mg/L**		**Iron deficiency defined as Ferritin<15μg/L or ferritin<70μg/L if CRP>5mg/L**		
	**Iron deficient N = 245**	**Iron replete N = 381**	**Iron deficient N = 319**	**Iron replete N = 307**	**Total N = 626**
Age (years)	24·5 (6·8)	20·3 (5·0)	23·4 (6·7)	20·4 (5·0)	22·0 (6·1)
Primigravid	87/245 (35·5%)	266/381 (69·8%)	138/319 (43·3%)	215/307 (70·0%)	353/626 (56·4%)
Gestational age (weeks)	22·4 (19·6-24·6)	21·9 (19·6-24·0)	22·4 (19·3-24·7)	21·9 (19·6-24·0)	22·0 (19·6-24·3)
Height (cm)	155·5 (6·8)	155·3 (6·4)	155·3 (6·5)	155·4 (6·6)	155·4 (6·5)
Weight (kg)	56·8 (8·8)	54·3 (7·8)	56·3 (8·7)	54·3 (7·7)	55·3 (8·3)
Body Mass Index (kg/m2) †	23·5 (3·3)	22·5 (2·8)	23·3 (3·3)	22·5 (2·7)	22·9 (3·0)
Religion ‡					
None	2/244 (0·8%)	0/379 (0·0%)	2/318 (0·6%)	0/305 (0·0%)	2/623 (0·3%)
Christian	174/244 (71·3%)	252/379 (66·5%)	225/318 (70·8%)	201/305 (65·9%)	426/623 (68·4%)
Muslim	67/244 (27·5%)	124/379 (32·7%)	90/318 (28·3%)	101/305 (33·1%)	191/623 (30·7%)
Other	1/244 (0·4%)	3/379 (0·8%)	1/318 (0·3%)	3/305 (1·0%)	4/623 (0·6%)
Education ‡					
None	2/237 (0·8%)	0/369 (0·0%)	2/310 (0·6%)	0/296 (0·0%)	2/606 (0·3%)
Lower Primary(1-5)	62/237 (26·2%)	80/369 (21·7%)	74/310 (23·9%)	68/296 (23·0%)	142/606 (23·4%)
Upper Primary(6-8)	87/237 (36·7%)	177/369 (48·0%)	124/310 (40·0%)	140/296 (47·3%)	264/606 (43·6%)
Lower Secondary(1-2)	29/237 (12·2%)	53/369 (14·4%)	41/310 (13·2%)	41/296 (13·9%)	82/606 (13·5%)
Upper Secondary(3-4)	48/237 (20·3%)	56/369 (15·2%)	60/310 (19·4%)	44/296 (14·9%)	104/606 (17·2%)
Tertiary	9/237 (3·8%)	3/369 (0·8%)	9/310 (2·9%)	3/296 (1·0%)	12/606 (2·0%)
Income source ‡					
None	17/244 (7·0%)	31/379 (8·2%)	22/318 (6·9%)	26/305 (8·5%)	48/623 (7·7%)
Subsistence farming	44/244 (18·0%)	81/379 (21·4%)	56/318 (17·6%)	69/305 (22·6%)	125/623 (20·1%)
Large scale farming	1/244 (0·4%)	1/379 (0·3%)	2/318 (0·6%)	0/305 (0·0%)	2/623 (0·3%)
Employed	38/244 (15·6%)	39/379 (10·3%)	45/318 (14·2%)	32/305 (10·5%)	77/623 (12·4%)
Casual work for wages	72/244 (29·5%)	136/379 (35·9%)	96/318 (30·2%)	112/305 (36·7%)	208/623 (33·4%)
Business	68/244 (27·9%)	88/379 (23·2%)	92/318 (28·9%)	64/305 (21·0%)	156/623 (25·0%)
Other	4/244 (1·6%)	3/379 (0·8%)	5/318 (1·6%)	2/305 (0·7%)	7/623 (1·1%)
HIV positive ‡	60/242 (24·8%)	28/377 (7·4%)	65/315 (20·6%)	23/304 (7·6%)	88/619 (14·2%)
Malaria RDT positive ¶	2/240 (0·8%)	7/372 (1·9%)	2/312 (0·6%)	7/300 (2·3%)	9/612 (1·5%)
Re-screened post positive malaria RDT §	20/245 (8·2%)	190/381 (49·9%)	56/319 (17·6%)	154/307 (50·2%)	210/626 (33·5%)
Venous Hb (g/dL)	8·80 (1·26)	8·84 (1·19)	8·83 (1·26)	8·82 (1·17)	8·82 (1·21)
Anaemia status (venous)					
No (Hb≥ 11 g/dL)	11/243 (4·5%)	19/380 (5·0%)	16/317 (5·0%)	14/306 (4·6%)	30/623 (4·8%)
Mild (10g/dL ≤ Hb < 11 g/dL)	25/243 (10·3%)	46/380 (12·1%)	33/317 (10·4%)	38/306 (12·4%)	71/623 (11·4%)
Moderate (7 g/dL ≤Hb <10 g/dL)	189/243 (77·8%)	294/380 (77·4%)	245/317 (77·3%)	238/306 (77·8%)	483/623 (77·5%)
Severe (Hb <7 g/dL)	18/243 (7·4%)	21/380 (5·5%)	23/317 (7·3%)	16/306 (5·2%)	39/623 (6·3%)
Ferritin (μg/L)	8·60 (6·20-12·40)	62·20 (36·30-119·80)	10·70 (6·80-26·80)	81·50 (36·30-140·70)	31·40 (10·50-79·30)

C-reactive protein (mg/L)	6·60 (3·50-10·60)	4·70 (2·50-11·20)	7·00 (4·30-12·30)	3·80 (2·10-8·40)	5·35 (2·90-11·00)
Inflammation *	152/245 (62·0%)	178/381 (46·7%)	226/319 (70·8%)	104/307 (33·9%)	330/626 (52·7%)

No·/total no· (%) are counts and percentages Plus-minus values are means ±SD· RDT denotes rapid diagnostic test (for *Plasmodium* parasitaemia), Hb haemoglobin, and HIV human immunodeficiency virus.
† Body mass index is the weight in kilograms divided by the square of the height in meters.
‡ Religion, Education, Income source, gravidity and HIV status were self-reported.
§ If women met the anaemia criteria but had a positive RDT, they were treated for malaria as per local protocols and deferred from enrolment. Women were invited to be re-screened – no earlier than seven days later – and be enrolled if they met the eligibility criteria (in these cases, parasitemia was assessed using microscopy due to persistence of antigen detection via RDT).
¶ Malaria RDT positive based on confirmatory RDT testing by lab personal on venous blood collected at enrolment.
* Inflammation indicates C-reactive protein>5mg/L.

Supplementary Table 4 · Baseline Characteristics excluding women who were malaria RDT-positive at screening, treated with AL and enrolled after microscopy-negative slide (re-screened) by iron status

	Iron deficiency defined as Ferritin<15μg/L or ferritin<30μg/L if CRP>5mg/L		Iron deficiency defined as Ferritin<15μg/L or ferritin<70μg/L if CRP>5mg/L		
	**Iron deficient N = 225**	**Iron replete N = 191**	**Iron deficient N = 263**	**Iron replete N = 153**	**Total N = 416**
Age (years)	24·8 (6·8)	21·4 (5·6)	24·2 (6·8)	21·5 (5·6)	23·2 (6·5)
Primigravid	76/225 (33·8%)	114/191 (59·7%)	100/263 (38·0%)	90/153 (58·8%)	190/416 (45·7%)
Gestational age (weeks)	22·6 (19·1-24·7)	22·1 (19·0-24·0)	22·6 (19·0-24·7)	21·9 (19·0-23·9)	22·3 (19·0-24·4)
Height (cm)	155·7 (7·0)	155·6 (7·1)	155·5 (6·9)	155·8 (7·3)	155·6 (7·0)
Weight (kg)	57·1 (9·0)	54·6 (8·2)	56·8 (8·9)	54·4 (8·2)	55·9 (8·7)
Body Mass Index (kg/m2) †	23·6 (3·4)	22·5 (3·1)	23·5 (3·4)	22·4 (3·0)	23·1 (3·3)
Religion ‡					
None	2/224 (0·9%)	0/189 (0·0%)	2/262 (0·8%)	0/151 (0·0%)	2/413 (0·5%)
Christian	160/224 (71·4%)	130/189 (68·8%)	189/262 (72·1%)	101/151 (66·9%)	290/413 (70·2%)
Muslim	61/224 (27·2%)	58/189 (30·7%)	70/262 (26·7%)	49/151 (32·5%)	119/413 (28·8%)
Other	1/224 (0·4%)	1/189 (0·5%)	1/262 (0·4%)	1/151 (0·7%)	2/413 (0·5%)
Education ‡					
None	1/217 (0·5%)	0/183 (0·0%)	1/254 (0·4%)	0/146 (0·0%)	1/400 (0·2%)
Lower Primary(1-5)	56/217 (25·8%)	45/183 (24·6%)	63/254 (24·8%)	38/146 (26·0%)	101/400 (25·2%)
Upper Primary(6-8)	79/217 (36·4%)	84/183 (45·9%)	96/254 (37·8%)	67/146 (45·9%)	163/400 (40·8%)
Lower Secondary(1-2)	26/217 (12·0%)	27/183 (14·8%)	32/254 (12·6%)	21/146 (14·4%)	53/400 (13·2%)
Upper Secondary(3-4)	46/217 (21·2%)	25/183 (13·7%)	53/254 (20·9%)	18/146 (12·3%)	71/400 (17·8%)
Tertiary	9/217 (4·1%)	2/183 (1·1%)	9/254 (3·5%)	2/146 (1·4%)	11/400 (2·8%)
Income source ‡					
None	17/224 (7·6%)	13/189 (6·9%)	17/262 (6·5%)	13/151 (8·6%)	30/413 (7·3%)
Subsistence farming	38/224 (17·0%)	43/189 (22·8%)	43/262 (16·4%)	38/151 (25·2%)	81/413 (19·6%)
Large scale farming	1/224 (0·4%)	0/189 (0·0%)	1/262 (0·4%)	0/151 (0·0%)	1/413 (0·2%)
Employed	35/224 (15·6%)	22/189 (11·6%)	41/262 (15·6%)	16/151 (10·6%)	57/413 (13·8%)
Casual work for wages	64/224 (28·6%)	57/189 (30·2%)	77/262 (29·4%)	44/151 (29·1%)	121/413 (29·3%)
Business	65/224 (29·0%)	52/189 (27·5%)	79/262 (30·2%)	38/151 (25·2%)	117/413 (28·3%)
Other	4/224 (1·8%)	2/189 (1·1%)	4/262 (1·5%)	2/151 (1·3%)	6/413 (1·5%)
HIV positive ‡	57/222 (25·7%)	21/189 (11·1%)	61/260 (23·5%)	17/151 (11·3%)	78/411 (19·0%)
Malaria RDT positive ¶	1/220 (0·5%)	3/186 (1·6%)	1/256 (0·4%)	3/150 (2·0%)	4/406 (1·0%)
Re-screened post positive malaria RDT §	0/225 (0·0%)	0/191 (0·0%)	0/263 (0·0%)	0/153 (0·0%)	0/416 (0·0%)
Venous Hb (g/dL)	8·79 (1·25)	8·94 (1·25)	8·83 (1·25)	8·91 (1·26)	8·86 (1·25)
Anaemia status (venous)					
No (Hb≥ 11 g/dL)	10/223 (4·5%)	15/190 (7·9%)	14/261 (5·4%)	11/152 (7·2%)	25/413 (6·1%)
Mild (10g/dL ≤ Hb < 11 g/dL)	23/223 (10·3%)	26/190 (13·7%)	28/261 (10·7%)	21/152 (13·8%)	49/413 (11·9%)
Moderate (7 g/dL ≤Hb <10 g/dL)	173/223 (77·6%)	137/190 (72·1%)	200/261 (76·6%)	110/152 (72·4%)	310/413 (75·1%)
Severe (Hb <7 g/dL)	17/223 (7·6%)	12/190 (6·3%)	19/261 (7·3%)	10/152 (6·6%)	29/413 (7·0%)

Ferritin (µ/L)	8·10 (6·20-11·90)	59·20 (33·70-123·10)	9·30 (6·40-15·70)	77·70 (27·10-139·80)	16·35 (7·85-52·55)
C-reactive protein (mg/L)	6·40 (3·40-10·50)	5·00 (2·70-11·40)	6·90 (3·80-11·70)	3·90 (2·30-9·00)	5·65 (3·10-11·00)
Inflammation *	138/225 (61·3%)	90/191 (47·1%)	176/263 (66·9%)	52/153 (34·0%)	228/416 (54·8%)

No·/total no· (%) are counts and percentages Plus-minus values are means ±SD· RDT denotes rapid diagnostic test (for *Plasmodium* parasitaemia), Hb haemoglobin, and HIV human immunodeficiency virus.
† Body mass index is the weight in kilograms divided by the square of the height in meters.
‡ Religion, Education, Income source, gravidity and HIV status were self-reported.
§ If women met the anaemia criteria but had a positive RDT, they were treated for malaria as per local protocols and deferred from enrolment. Women were invited to be re-screened – no earlier than seven days later – and be enrolled if they met the eligibility criteria (in these cases, parasitemia was assessed using microscopy due to persistence of antigen detection via RDT).
¶ Malaria RDT positive based on confirmatory RDT testing by lab personal on venous blood collected at enrolment
* Inflammation indicates C-reactive protein>5mg/L.

Supplementary Table 5. Comparison by WHO definition of iron status at baseline and qPCR positivity

	**Iron deficient at baseline**		**Iron replete at baseline**		**Unadjusted***		**Adjusted***	
**PCR positive**	**N**	**n/N (%)**	**N**	**n/N (%)**	**Effect size (95% CI)**	**p-value**	**Effect size (95% CI)**	**p-value**
Baseline*	319	92/319 (28·8)	307	186/307 (60·6)	0·48 (0·37, 0·611)	<0·0001	0·62 (0·47, 0·81)	0·0006
28 days post treatment+	336	50/336(14.9)	307	65/307(21.2)	0·71(0·49, 1.02)	0.064	0·87 (0·56, 1.34)	0.52
Excluding those malaria RDT-positive at screening, treated with AL and enrolled after microscopy-negative slide (re-screened)								
Baseline*	263	61/263 (23.2)	153	88/153 (54.6)	0·40 (0·29, 0·56)	<0·0001	0·49 (0·35, 0·70)	<0·0001
28 days post treatment+	279	39/279 (14.0)	152	35/152 (23.0)	0·61 (0·38, 0·96)	0.032	0·75 (0·45, 1·25)	0·27

Iron deficient indicates serum ferritin <15 µg/L or ferritin <70 µg/L if C-reactive protein >5 mg/L

*Unadjusted: N/A;

Adjusted: gestational age at baseline, gravidity status, HIV, rescreened + AL treatment, maternal age, education, income source, and religion

+Unadjusted: treatment group:

Adjusted: treatment group, gestational age at baseline, gravidity status, HIV, rescreened + AL treatment, maternal age, education, income source, and religion

*Poisson regression model with robust error variance was fit

Supplementary Table 6. Prevalence of qPCR-positivity at 28 days post treatment in qPCR-negative women at baseline by iron status

	Iron deficient at base		Iron replete at base		Unadjusted *		Adjusted*	
**PCR positive**	**N**	**n/N (%)**	**N**	**n/N (%)**	**Effect size (95% CI)**	**p-value**	**Effect size (95% CI)**	**p-value**
28 days post treatment+	215	16/215 (7·4)	177	20/177 (11·3)	0·66 (0·34, 1·27)	0·21	0·95 (0·41, 2·19)	0·91

Unadjusted: N/A;
adjusted: Gestational age at baseline, gravidity status, HIV, rescreened + AL treated, maternal age, education, income source, religion
+Unadjusted: treatment group:
+Adjusted: treatment group, gestational age at visit 2, gravidity, HIV, rescreened + treated, maternal age, education, income source, religion
*Poisson regression model with robust error variance was fit

Supplementary Table 7. Parasite densities at 28 days post treatment in qPCR-negative women at baseline, by iron status at baseline

	**Iron deficient at baseline**	**Iron replete at baseline**	**Overall**
	N=16	N=20	N=36
parasites per mL of blood	3·27 (0·70-439·66)	19·60 (0·33-558·50)	4·48 (0·35-522·32)
Log_e parasites per mL of blood	1·18 (-0·49-6·00)	2·97 (-1·11-6·30)	1·48 (-1·04-6·24)

Values represent the median and IQR.

Due to small sample sizes and violation of assumptions, only summary data are presented for the continuous parasite densities without statistical analysis comparing groups.

Supplementary Table 8. List of Primers and Probes used in this study.

Species	Gene	Forward sequence	Reverse sequence
Mus Musculus (C57BL6)	*Hamp*	CAGGGCAGACATTGCGATAC	TGCAACAGATACCACACTGGG
Mus Musculus (C57BL6)	*Hrpt*	ACAGGCCAGACTTTGTTGGA	ACTTGCGCTCATCTTAGGCT
Mus Musculus (C57BL6)	*Tmprss6*	TCACCTCGGTCTATGGGTGC	CCCTCCAGTGTCAGGTTCAC
Mus Musculus (C57BL6)	*Gapdh*	GGAGAGTGTTTCCTCGTCCC	ATGAAGGGGTCGTTGATGGC
Plasmodium falciparum (3D7)	*Pf-varATS*	CCCATACACAACCAAYTGGA	TTCGCACATATCTCTATGTCTATCT
Plasmodium falciparum (3D7)	*Pf-varATS-probe*	FAM-TRTTCCATAAATGGT-NFQ-MGB	
Plasmodium falciparum (3D7)	*Pf-18SrRNA DNA*	TATTGCTTTTGAGAGGTTTTGTTG	ACCTCTGACATCTGAATACGAATGC
Plasmodium falciparum (3D7)	*Pf-18SrRNA-probe*	FAM-ACGGGTAGTCATGATTGAGTT-MGB-NFQ	

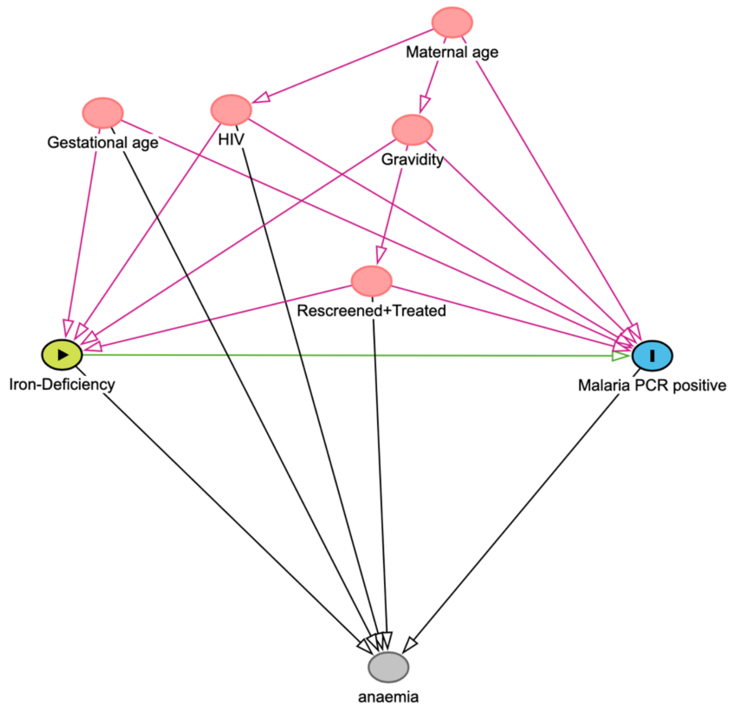

Supplementary figure 1. Directed Acyclic Graph of the association between Iron-deficiency and Plasmodium qPCR-positivity at trial baseline. Arrows indicate the direction of the assumed causal influence. The primary exposure of interest is iron-deficiency status at baseline (green node) and the outcome is *Plasmodium* infection detected by qPCR at baseline (blue node). Measured variables that influence both the exposure and the outcome are shown (red nodes). Variables not adjusted for in the main analysis are indicated in grey. This DAG was constructed in DAGitty (http://www.dagitty.net) based on existing literature and subject-matter knowledge to guide variable selection for the minimal adjustment set to obtain the total effect and identify potential sources of bias.

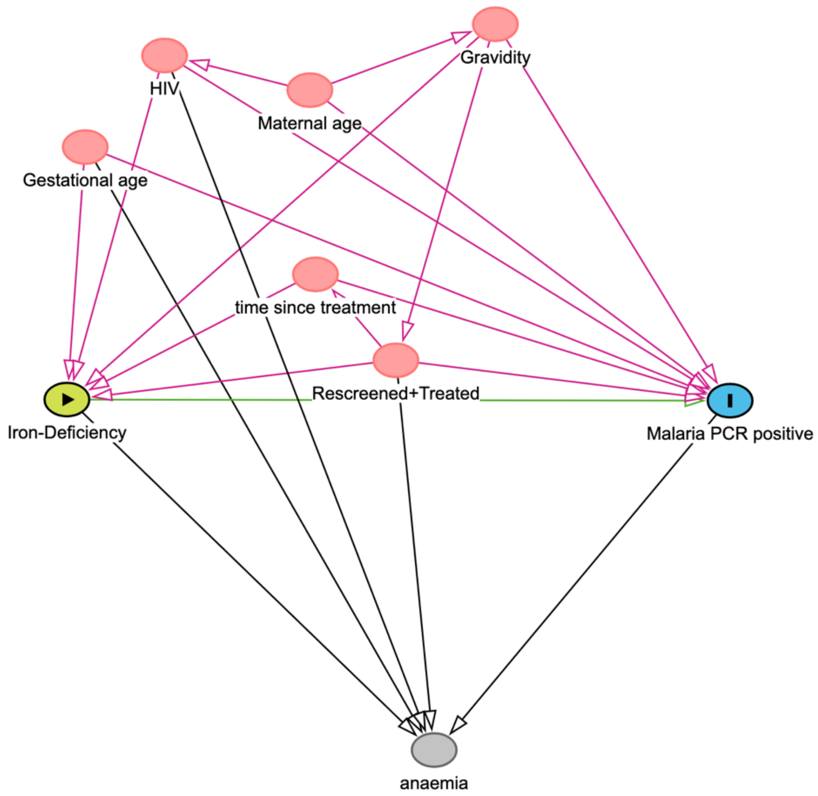

Supplementary figure 2. Directed Acyclic Graph of the association between Iron-deficiency and Plasmodium qPCR-positivity at 28 days post treatment. Arrows indicate the direction of the assumed causal influence. The primary exposure of interest is iron-deficiency status at baseline (green node) and the outcome is *Plasmodium* infection detected by qPCR at 28 days post treatment (blue node). Measured variables that influence both the exposure and the outcome are shown (red node). Variables not adjusted for in the main analysis are indicated in grey. This DAG was constructed in DAGitty (http://www.dagitty.net) based on existing literature and subject-matter knowledge to guide variable selection for the minimal adjustment set to obtain the total effect and identify potential sources of bias.

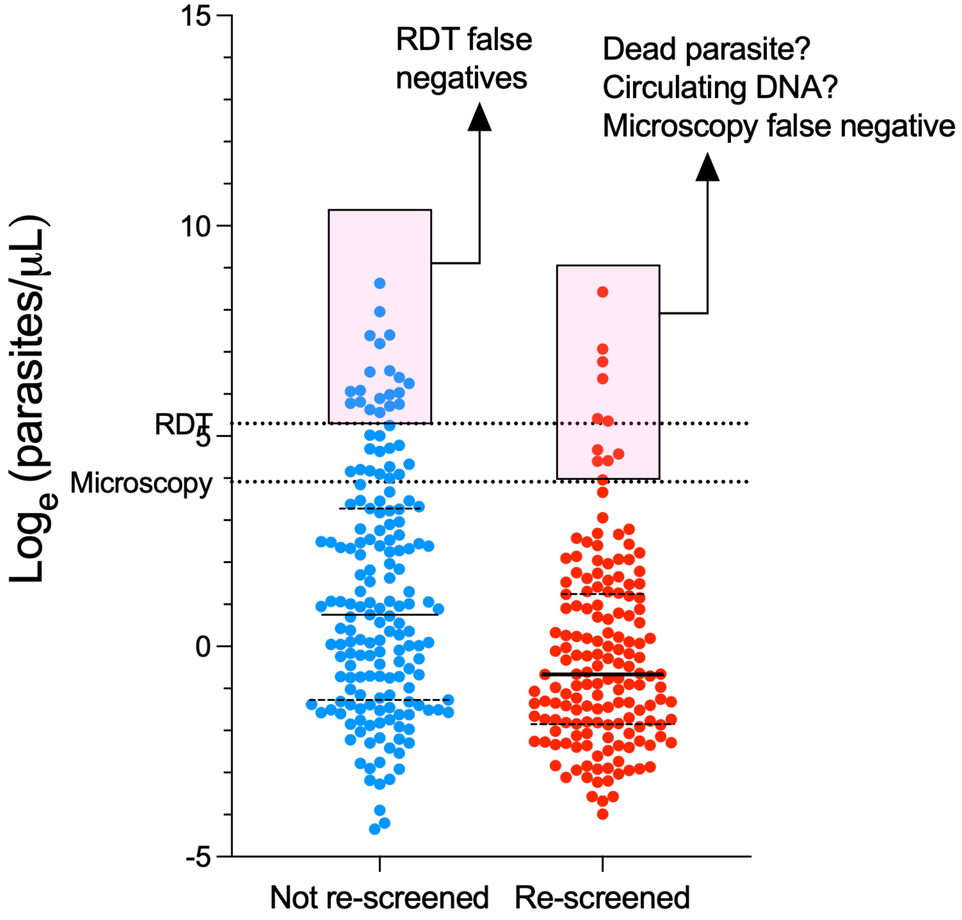

Supplementary figure 3. P falciparum density by screening status. Parasite density as measured by ultrasensitive qPCR (log_e parasites/mL) in women at baseline, comparing those who entered the study directly (not re-screened (blue)) with those who entered the study after being initially RDT-positive at the time of screening, treated with artemether-lumefantrine, and allowed to be re-screened and enrolled if they had a negative microscopy slide (Re-screened (red)). Dotted lines represent the theoretical limits of detection for malaria RDTs (200 parasites/μL) and microscopy slides (50 parasites/μL).

A

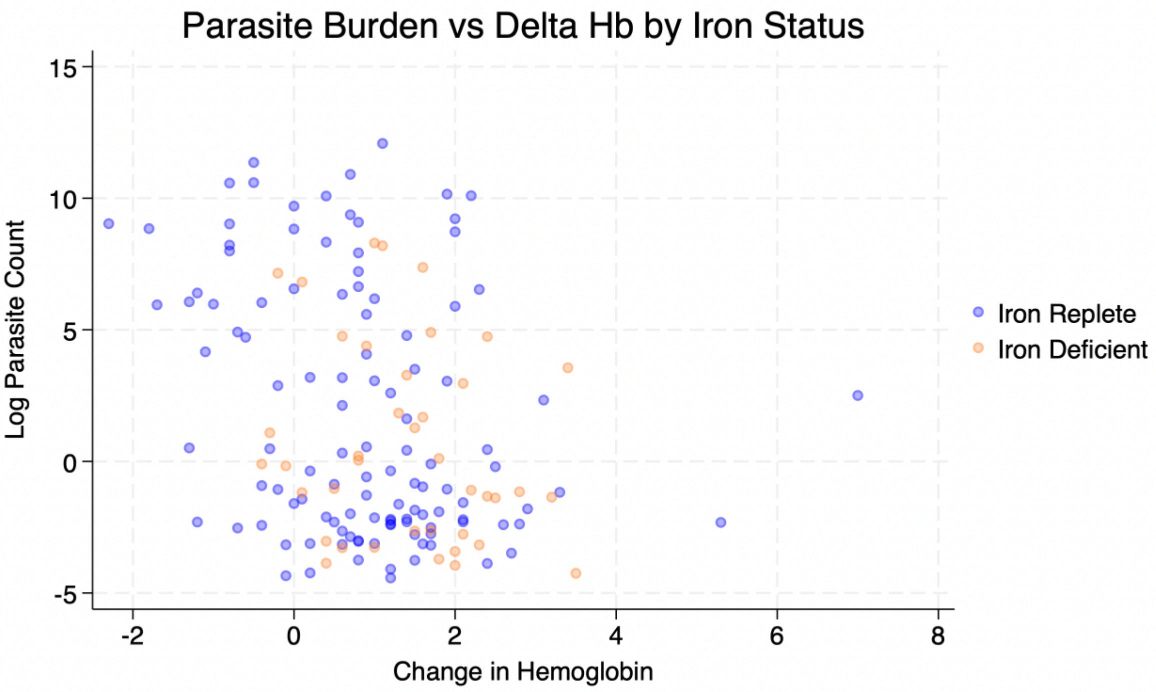

B

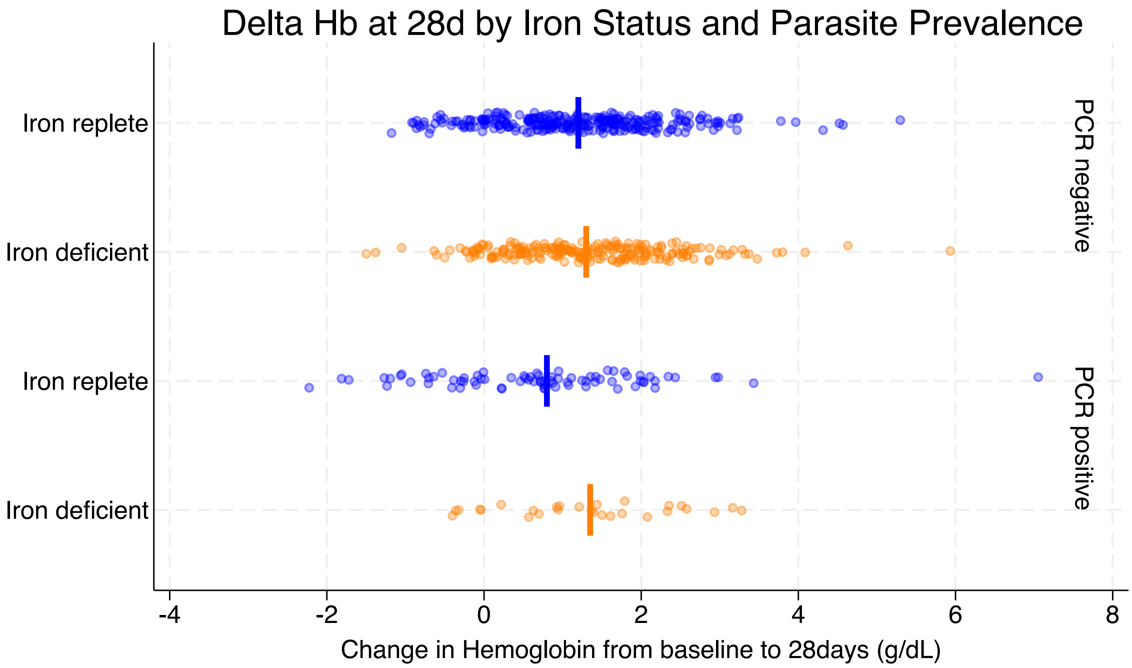

Supplementary figure 4. Parasite burden and hemoglobin response by iron status A. Parasite burden vs Delta Hb by Iron Status. Parasite density as measured by ultrasensitive qPCR (log_{10} parasites/mL) in women at 28 days post-treatment vs hemoglobin response at 28 days post-treatment, measured as change in hemoglobin from baseline to day 28, stratified by baseline iron status (Iron replete, blue; Iron deficient, orange). B. Delta Hb at 28d by Iron Status and Parasite Prevalence. Change in hemoglobin from baseline to 28 days post-treatment (g/dL) stratified by

baseline iron status (Iron replete, blue; Iron deficient, orange) and parasite prevalence at 28 days as determined by ultrasensitive qPCR (PCR negative, top panels; PCR positive, bottom panels). Lines represent median.

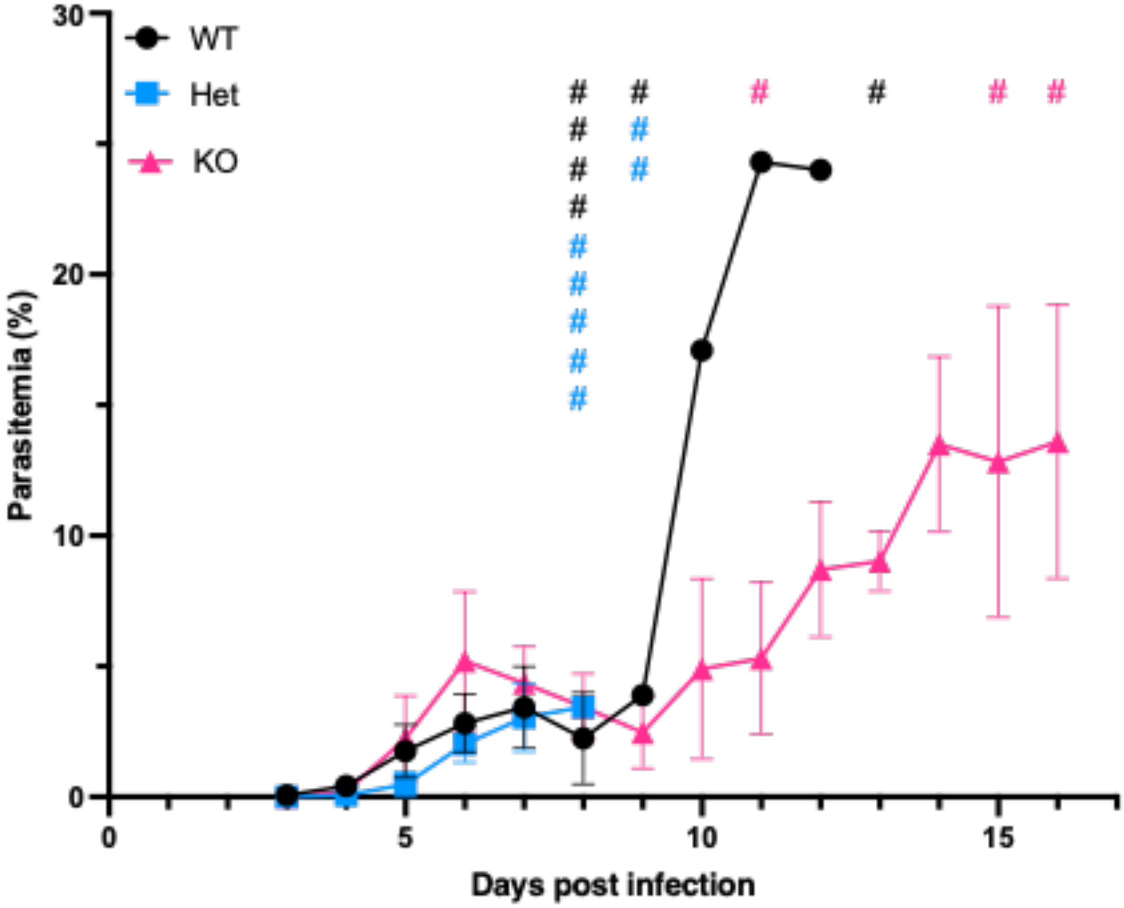

Supplementary figure 5. Blood-stage parasitaemia up to day 16 post infection. *P. berghei* blood-stage parasitaemia in C57BL/6 wild-type (WT), *Tmprss6*-heterozygous (Het), and *Tmprss6*-knockout (KO) mice. Data represented as mean ± SD, n=6-7 mice per group. # = euthanasia.
